# Social Prescription Interventions Addressing Social Isolation and Loneliness in Older Adults: Meta-Review Integrating On-the-Ground Resources

**DOI:** 10.2196/40213

**Published:** 2023-05-17

**Authors:** Catherine Paquet, Jocelyne Whitehead, Rishabh Shah, Alayne Mary Adams, Damion Dooley, R Nathan Spreng, Anna-Liisa Aunio, Laurette Dubé

**Affiliations:** 1 Département de Marketing Faculté des Sciences de l'Administration Université Laval Québec, QC Canada; 2 Centre de Recherche, Centre Hospitalier Universitaire de Québec - Université Laval Quebec, QC Canada; 3 Centre Nutrition, Santé et Société (NUTRISS), Institut sur la Nutrition et les Aliments Fonctionnels (INAF) Quebec, QC Canada; 4 Integrated Program in Neuroscience McGill University Montreal, QC Canada; 5 Desautels Faculty of Management McGill University Montreal, QC Canada; 6 McGill Centre for the Convergence of Health and Economics McGill University Montreal, QC Canada; 7 Department of Family Medicine McGill University Montreal, QC Canada; 8 Centre for Infectious Disease Genomics and One Health Simon Fraser University Vancouver, BC Canada; 9 Department of Neurology and Neurosurgery McGill University Montreal, QC Canada; 10 Department of Sociology Dawson College Montreal, QC Canada

**Keywords:** social prescription, social isolation, loneliness, intervention, older adults, knowledge mobilization, database management, ontology

## Abstract

**Background:**

Social prescription programs represent a viable solution to linking primary care patients to nonmedical community resources for improving patient well-being. However, their success depends on the integration of patient needs with local resources. This integration could be accelerated by digital tools that use expressive ontology to organize knowledge resources, thus enabling the seamless navigation of diverse community interventions and services tailored to the needs of individual users. This infrastructure bears particular relevance for older adults, who experience a range of social needs that impact their health, including social isolation and loneliness. An essential first step in enabling knowledge mobilization and the successful implementation of social prescription initiatives to meet the social needs of older adults is to incorporate the evidence-based academic literature on what works, with on-the-ground solutions in the community.

**Objective:**

This study aims to integrate scientific evidence with on-the-ground knowledge to build a comprehensive list of intervention terms and keywords related to reducing social isolation and loneliness in older adults.

**Methods:**

A meta-review was conducted using a search strategy combining terms related to older adult population, social isolation and loneliness, and study types relevant to reviews using 5 databases. Review extraction included intervention characteristics, outcomes (social [eg, loneliness, social isolation, and social support] or mental health [eg, psychological well-being, depression, and anxiety]), and effectiveness (reported as consistent, mixed, or not supported). Terms related to identified intervention types were extracted from the reviewed literature as well as descriptions of corresponding community services in Montréal, Canada, available from web-based regional, municipal, and community data sources.

**Results:**

The meta-review identified 11 intervention types addressing social isolation and loneliness in older adults by either increasing social interactions, providing instrumental support, promoting mental and physical well-being, or providing home and community care. Group-based social activities, support groups with educational elements, recreational activities, and training or use of information and communication technologies were the most effective in improving outcomes. Examples of most intervention types were found in community data sources. Terms derived from the literature that were the most commonly congruent with those describing existing community services were related to telehealth, recreational activities, and psychological therapy. However, several discrepancies were observed between review-based terms and those addressing the available services.

**Conclusions:**

A range of interventions found to be effective at addressing social isolation and loneliness or their impact on mental health were identified from the literature, and many of these interventions were represented in services available to older residents in Montréal, Canada. However, different terms were occasionally used to describe or categorize similar services across data sources. Establishing an efficient means of identifying and structuring such sources is important to facilitate referrals and help-seeking behaviors of older adults and for strategic planning of resources.

## Introduction

### Background

As in other nations, there is an increasing demand in Canada to incorporate social determinants of health (SDOH) into health assessments and address the social needs of citizens through primary care [1-6]. Evidence suggests that SDOH have sizable effects on disease onset and progression [7,8] and that greater spending on social services and public health is associated with improvements in health outcomes such as lower prevalence rates of obesity, asthma, lung cancer, acute myocardial infarction, and type 2 diabetes [9]. In the wake of COVID-19, health systems are grappling with the disproportionate impact of the pandemic on low-income, minority, and other vulnerable populations [10]. Currently, patient care and health information exist in silos across primary care, communities, and public health organizations, where the lack of coordination and limited flow of information hampers service access to those most in need [11]. Although medical and nonmedical issues are often intricately coupled, primary care providers are not always equipped to address SDOH and must rely on community resources to provide alternative personalized care and support [12]. Therefore, there is an urgent need for the synthesis and integration of knowledge surrounding SDOH and related evidence-based interventions. However, appropriate, whole-person care for patients is a function of the availability and accessibility of such interventions within the community and the ability of health professionals to draw from these resources.

To address these challenges, social prescription programs that enable primary care providers to refer patients to a range of local nonmedical services, including activities or resources provided by the local voluntary and community sectors, are receiving increasing attention [13]. Such programs not only have the capacity to provide personalized care but are also an effective solution for addressing lifestyle-related health concerns and nonbiomedical issues associated with SDOH [14]. Nevertheless, the intensive and time-consuming case management required for such coordination presents a significant challenge to such initiatives [15]. Connectors or link workers have provided a temporary solution, offering the knowledge, skills, and resources required to partner patients with services [16]. However, as interventions advance, services expand, and patients seek increasingly personalized care, the role of link workers will become increasingly complex and labor intensive.

To facilitate this process, digital social prescription tools have surfaced, using a related approach to seamlessly integrate services of diverse, multisectoral organizations (often through different systems) and deliver high standards of interoperability. For example, Evergreen Life cloud software (Evergreen Solutions Ltd) [17] offers access to an in-app virtual link worker, where automated features allow for self-referral by linking medical records to a database of nonmedical services. Similarly, Egton Medical Information Systems (EMIS) Health’s Elemental Social Prescription Connector (EMIS Group) [18] is a digital platform integrated into the computers of primary care providers, co-designed by local councils, community and voluntary sector organizations, and academic institutions, among others. These initiatives demonstrate promises in helping organize collaboration for improving social care delivery. However, their practical application and effectiveness rely on the development and maintenance of a comprehensive, up-to-date inventory of services that capture on-the-ground activities that address the specific social need initially identified. Likewise, the absence of a standardized approach for identifying and categorizing available services complicates their adaptation to other contexts.

The key enablers of these digital initiatives are ontological technologies, that is, knowledge organization systems that demonstrate the properties of a concept and the relationships that exist between those properties. Ontologies are an important tool for establishing a comprehensible knowledge network for managing data, enabling inferences on the relationship of said data, and promoting interoperability across data systems [19,20]. As dynamic entities, ontologies are likely to evolve alongside ongoing services [21] and require updating to reflect the incorporation of new evidence [22]. They provide a standardization for data entry and retrieval, are machine processable, and thus offer the potential for automation [23]. User-centered ontologies, specifically, provide a controlled vocabulary common to and agreed upon by a diverse set of stakeholders, thereby optimizing the interaction of users with the expert system [20]. These conceptual frameworks typically include additional information such as synonyms and use examples as well as the relationship between entities, which are often more intricate and evolved than a simplistic hierarchical structure [22]. Incorporating artificial intelligence and machine learning techniques provides the means for real-time maintenance and updating of databases and can assist to optimally match patient needs to suitable services. Placed within the social prescription context, the development of an expressive ontology for organizing knowledge and resources could translate to a seamless navigation of diverse community interventions and services made accessible to the end user—whether patient, health care provider, policy maker, or researcher—based on identified needs. Investments are currently underway to standardize information related to social prescription and social determinants in an effort to facilitate their integration of such information into electronic health records [24,25]. However, these efforts are disproportionately concentrated on the identification of the social needs and less so on the classification and characterization of resources that could be used to address such needs.

### The Case of Social Isolation and Loneliness

Extended social isolation and the experience of loneliness during the pandemic have been detrimental to people’s well-being [26], marking an urgent need to mitigate social and mental health consequences [27]. Older adults are especially susceptible to social isolation and loneliness, which are often associated with adverse health outcomes such as depressive symptomatology [28,29], cognitive [30-32] and functional decline [33], cardiovascular disease [34], and an increased risk of mortality [35]. The relationship between social isolation and mortality has been compared in magnitude with that of traditional risk factors (smoking and high blood pressure) [36]. As the percentage of individuals aged >65 years will nearly double over the next 30 years [37], the need for evidence-informed guidance is even more pressing.

Although remote and in-person interventions have been proposed to reduce the impact of social isolation and to promote social connectedness in older adults [38,39], their implementation is often complex. A particular challenge pertains to matching services to patients’ specific needs, given the need for input and coordination from a diverse set of siloed stakeholders [40]. Novel social prescription programs have the potential to bridge this gap, as primary care providers are well positioned to make referrals to local nonmedical services [13,16].

This study integrates scientific evidence, collected through an extensive meta-review, with on-the-ground evidence on real-world intervention, initiating the development of a social prescription ontology using social isolation and loneliness of older adults as a use case. The meta-review explores effective interventions for improving social isolation and loneliness in older adults and mitigating their impact on well-being, resilience, and coping. Concordance across intervention terms extracted from the literature and those identified within local community web-based resources of a sample population (Montréal, Canada) was explored. The cross-comparison of terms and initial syntactic integration (knowledge transfer) will provide the foundation for developing a social prescription ontology, orchestrated through supplementary phases of semantic integration (knowledge translation) and pragmatic integration (knowledge transformation [41]). Importantly, we explored technology-enabled social prescribing as a viable approach to building a sustainable and scalable way of delivering interventions that are both precise and adaptable [14]. A more detailed and representative ontology for social prescription has the potential to provide more precise and accurate insight into the attributable outcomes of social prescribing [42].

## Methods

### Meta-Review

The search strategy entailed a review of reviews (meta-review) of studies reporting on social isolation and loneliness in older adults, developed in collaboration with an academic librarian and in accordance with the PRISMA (Preferred Reporting Items for Systematic Reviews and Meta-Analyses) guidelines [43]. The search strategy (outlined in Multimedia Appendix 1) combined terms related to the (1) older adult population, (2) social isolation and loneliness, and (3) study types relevant to reviews, using the following databases: MEDLINE, Embase, PsycINFO, Social Work Abstracts, and CINAHL. Reviews were excluded if they did not clearly report on interventions directly related to social isolation or loneliness, focused on institutional settings only (eg, nursing homes) or caregivers, did not use a systematic search strategy [44], were not focused on an older adult population (defined as aged 50 years for the purpose of the review), did not include a full description of the study results (abstracts and protocols), were not published in French or English, or were published >20 years ago from the time of extraction (July 2020). No restrictions were imposed on the type of evidence reviewed within the reviews. Screening was performed in 2 steps by 2 reviewers, and discrepancies were resolved through discussion. As part of the larger project, extracted reviews reporting on interventions of social isolation or loneliness as well as risk and protective factors were selected [45]; the latter is beyond the scope of this study and is only briefly addressed in the *Discussion* section.

### Data Extraction

Extraction tools were developed and piloted before use. Extractions were made by a single reviewer, whereas a random selection of 10% of reviews was systematically verified by another reviewer. The information extracted included the context, outcome, design of the studies reviewed, type of review, years covered, number of studies included, population, type of interventions reviewed, limitations of the studies reviewed, and conclusions. Select items from a quality appraisal tool [46] were used, related to the justification of the study design, completeness of the search strategy, duplication of study selection and extraction stages, appropriate discussion of included studies, and inclusion of a quality appraisal (Multimedia Appendix 2 [38,47-95]). The interventions were classified into groups of similar types, which were then grouped into overarching themes based on their intended outcomes.

To provide an accurate synthesis of information related to a given type of intervention, eligible primary studies (quantitative and qualitative) falling within each type of intervention were identified from the reviews. Following the removal of duplicates, the following information was extracted from the primary studies: design type, intervention mode of delivery (in person or on the web), effect size (where available) and level of intervention (individual, group, or community), duration and frequency, organizations or professional body involved, quantitative and qualitative findings, presence of moderating or mediating factors, and undesirable consequences (refer to the study by Paquet et al [45]). Given the meta-review nature of the synthesis, effectiveness was based on the information reported in the reviews, which often provided limited information on effect size. Therefore, effectiveness was categorized into the following: (1) reported effect consistent with effectiveness, (2) reported effect providing mixed evidence for effectiveness (eg, trend toward significant effect, effect for subpopulation only, and a qualitative self-reported effect), and (3) reported effect not supporting effectiveness. Any discrepancies across reviews, primarily related to calculated effect sizes from meta-analyses, were indicated (*) in accordance with the multimedia appendices of the study by Paquet et al [45]. Effectiveness was compiled across intervention types and reported separately for social (eg, changes in social isolation, felt loneliness, social networks or connections, social support or social communication or participation) and mental health (eg, changes in mental health and well-being, depression, or anxiety) outcomes.

### Community Resource Identification and Comparison of Intervention Terms

The investigation of available “on-the-ground” community interventions or services occurred with a focus on 2 boroughs of the city of Montréal, which offer access to a culturally diverse population, serving a mostly English-speaking or bilingual (French-English) population [96] and where social prescription efforts are being developed [97]. Montréal has Québec’s largest number of older adults living in poverty and lacking social support, where the proportion of older adults living alone by borough ranges from 22% to 57% [98]. Two researchers independently extracted intervention terms from the meta-review corresponding to each type of intervention identified. The terms extracted by both the reviewers were included.

### Data Sources

A finalized list of extracted intervention terms was used to search for and catalog corresponding community services for reducing social isolation and loneliness in directories of municipal and community resources, as well as websites of nonprofit organizations that serve older adults within the Montréal community (referred to hereafter as “data sources”). Data sources were included if the services offered matched the description of ≥1 intervention types identified during the meta-review process. Table 1 outlines the web-based data sources used, a description of the sources, and how each source was used to identify relevant intervention terms.

**Table 1:** Community data source identification and description.

Data source and description			Identification and use of the relevant terms
**Regional or municipal**			
	211 Grand Montréal [99]: web-based and phone service aiming to connect citizens to community organizations and services within Greater Montréal. The database contains detailed contact, location, and services information about public, para-public, and community organizations that may serve the “senior” population.	The database was filtered for “seniors,” “Montréal” location, and “English” as the service language. The output included a list of subcategories containing pertinent community organizations, of which some overlapped across subcategory offering a broad range of services. The organizations with service descriptions relevant to reducing loneliness or social isolation were identified, and keywords were extracted from these descriptions and keyword tags.	
	Données Montréal [100]: open-access data to services and events provided by the city of Montréal and offered within the Montréal area, several of which have the potential to improve mental well-being. Données Montréal data sets are not necessarily specific to older adults, and they do not explicitly state interventions or services to reduce loneliness and isolation.	Nine data sets were extracted from Données Montréal owing to their potential for providing information on infrastructure aimed at reducing loneliness and isolation in Montréal. Only databases that offered information regarding ≥1 intervention types were extracted. Four of these data sets were relevant, providing information on cultural activities, lists of parks, seasonal recreational activities, and museums, libraries, or recreational activities in Montréal. The data sets were reported in French and were translated into English for the extraction. Each data set varied with respect to how the information or events were reported. For example, some provided geolocations, time, and events, whereas others offered description of events. Keywords relating to events or places that one could visit for reducing loneliness and social isolation were extracted. None of the events and places were specific to older adults but were evidently accessible to them and were primarily related to recreational activities.	
**Neighborhood-specific resources**			
	Centraide of Greater Montréal [101]: directory of community agencies in Montréal, as separated by boroughs, that receive financial support from the Centraide of Greater Montréal Foundation. Centraide aims to reduce poverty and exclusion in the city of Montréal and funds community organizations to achieve this objective. Two specific neighborhoods or boroughs in Montréal were investigated, Côte-des-Neiges and Notre-Dame-de-Grace, where organizations serving older individuals with services relevant for reducing loneliness and social isolation were included.	The directory of supported agencies and projects were searched on the Centraide website. The 2 target boroughs were searched for through the filter “Territory served,” providing a list of organizations that Centraide supports specific to these regions. Each organization had a list of activities offered, where activities that compared with those identified in the meta-review for reducing loneliness and isolation and accessible to older adults were extracted.	
	Community outreach worker: a comprehensive list of community organizations available in the region of Notre-Dame-de-Grace, as collected by a community outreach worker for the same period. Several organizations offer services aimed at reducing loneliness and social isolation and supporting older adults in the community.	The database was obtained from the community outreach worker, and relevant organization services were identified that reflected the meta-review intervention types. Keywords were extracted from the service descriptions, as formulated by the outreach worker.	
**Population-specific resources (older adults)**			
	Contactivity Centre [102]: a community center (nonprofit) serving older adults in Montréal that offers resources, referrals, and activities to improve the mental, emotional, and physical well-being of its members. It maintains a large database of organizations that offer a variety of services.	The “Resource and referrals” page of the Contactivity Centre website provides categories of services offered, listing community organizations and the corresponding websites that can provide access to those services. The relevant categories identified included “Recreational and Community Services,” “Friendly Visits,” “Daily Phone Calls,” and “Senior Advocacy.” Under each category, the listed organization websites were searched, and keywords were extracted from the services that compared with those identified in the meta-review.	
	AMI-Québec [103]: a nonprofit organization providing access to courses, workshops, interest groups, activities, and services in person or on the web as well as a database of external community organizations providing comparable services for the older adult population. Some of the external organizations are specific to older adults and offer services to reduce loneliness and social isolation.	The “Resource List” on the AMI-Québec website included a “Special Populations” category and a “Seniors” subcategory, which listed relevant community organizations with a short description of services offered. These lists were searched and relevant keywords were extracted from the services descriptions.	
	FADOQ^a^ [104]: The largest older adult organization in Canada, whereby membership provides access to hundreds of clubs and programs designed for older adults.	FADOQ is a network of older adult clubs and groups promoting active and healthy aging. They offer programs and activities related to recreational, physical, and ICT^b^ intervention types through their clubs. Information available from their website was mostly about individual clubs, web-based resources, and provincial older adult events.	
	RIAQ^c^ [105]: a nonprofit Québec Seniors Information Network, run by older adults, to offer members knowledge resources, workshops, and services related to information technology, health, and travel.	RIAQ is an information network for older adults and offers workshop in technology-related fields, which were identified as relevant for reducing loneliness and isolation through the ICT intervention type.	

^a^FADOQ: Réseau Fédération de l’âge d’or du Québec.

^b^ICT: information and communication technology.

^c^RIAQ: Réseau d’information des aînés du Québec.

Terms were primarily identified within the description of services provided by the community organizations or from a list of services keywords (if one was provided) available through the data source. If information regarding an organization’s services was not provided through the data source, the organization’s website was searched, and keywords were extracted from the relevant service descriptions. As Données Montréal [100], Réseau Fédération de l’âge d’or du Québec (FADOQ) [104], and Réseau d’information des aînés du Québec (RIAQ) [105] did not list community organizations, keywords were instead identified based on the activities and services listed. Intervention keywords were only extracted if they described activities, services, or infrastructure that supported the reduction of loneliness or social isolation and if they were relevant for or directed to older adults. Terms that fell under an intervention type but were not extracted from the meta-review were reported as “terms present in community resources only.”

## Results

### Meta-Review Search Outcomes

**Figure 1:**
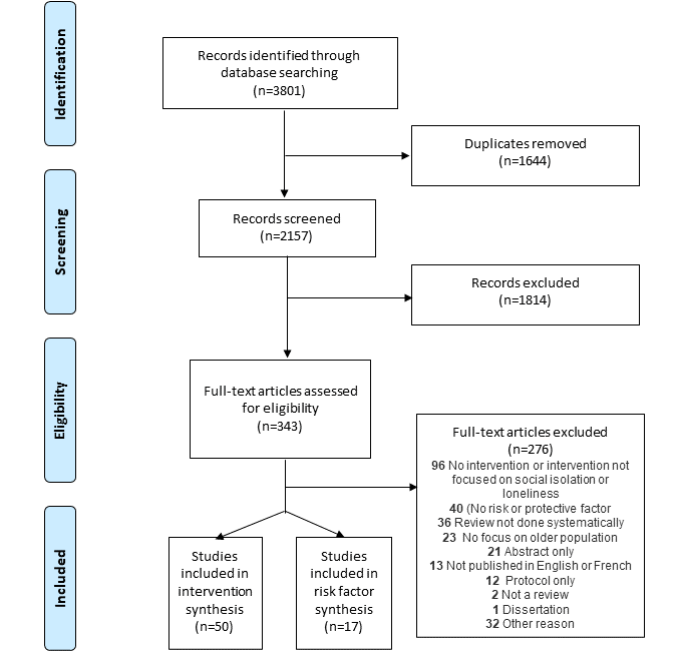
Study selection process.

A total of 3801 reviews were identified (MEDLINE: n=1012, Embase: n=1383, PsycINFO: n=664, Social Work Abstracts: n=5, and CINAHL: n=737). After removing duplicates, 2157 studies were screened based on abstract and title. Of the 2157 reviews, 343 (15.9%) full-text reviews were assessed for eligibility. Of 343 reviews, 50 (14.6%) met the eligibility criteria as interventions to reduce social isolation or loneliness (Figure 1).

### Characteristics of the Included Studies

Included reviews covered publications up to 2018. The reviews varied in quality, as reflected by the number of the selected AMSTAR (A Measurement Tool to Assess Systematic Reviews) criteria met by each review (Multimedia Appendix 2). The criteria that were less often met were double extraction of information (17/50, 34% of reviews) and assessment (24/50, 48%) and consideration of risk of bias (25/50, 50%).

### Intervention Type and Characteristics

#### Overview

**Figure 2:**
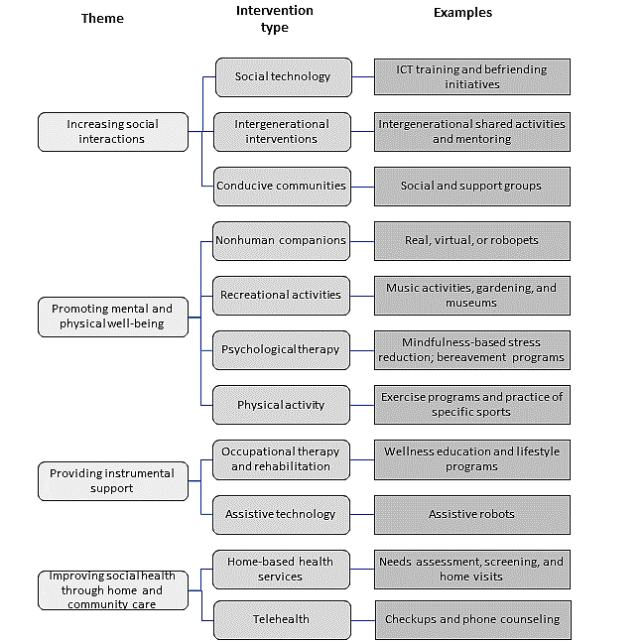
Representation of the identified intervention types (with examples) and their associated themes. ICT: information and communication technology.

Eleven intervention categories were identified that were grouped into four themes reflecting their objectives: (1) *increasing social interactions*, including social technology, intergenerational interventions, and conducive communities; (2) *promoting mental and physical well-being*, including nonhuman companions, recreational activities, psychological therapy, and physical activities; (3) *providing instrumental support*, that is, offering lifestyle changes through occupational therapy (OT) or rehabilitation or assistive technology to target sources of social-based deficiencies; and (4) *providing home and community care*, including home-based health services and telehealth, which are primarily one-on-one interventions provided by a regulated health care professional, based outside of an institutional setting, and aimed at directly or indirectly addressing social care needs. The division of the 11 intervention categories generally reflected the approach taken within the reviews themselves. Some refinement was conducted to minimize the overlap of categories (ie, reduced redundancy of intervention term or keyword extracts) and ensure that our analyses had sufficient granularity to produce informative insights. The 11 intervention categories and their respective themes along with selected examples are shown in Figure 2. Information related to interventions under each theme is presented in Multimedia Appendix 3 and is summarized in the following section.

#### Increasing Social Interactions

Interventions with the intended outcome of increasing social interactions functioned through developing opportunities and providing platforms where new relationships could be formed and social networks could flourish. The first type of intervention was intergenerational interventions that aimed at connecting older adults with youth to establish valuable partnerships and mutual responsibility through shared (1) recreational activities [106-116]; (2) activities led by older adults, such as those incorporating education and skills development [117-121], reading activities [122-125] or mentorship [126,127]; and (3) therapy-focused interventions, such as reminiscence therapy [128-130]. The second type of intervention under this theme aimed to provide older adults with a virtual space to foster new relationships and reinforce existing ones (social technology). These interventions were primarily composed of information and communication technology (ICT) training or networking sessions with older adults [131-149] as well as telephone or video call befriending initiatives [150-156] or radio programs [157], most commonly taking place at home on a weekly basis. The final type of intervention under this theme aimed to provide older adults access to supportive and conducive environments or communities (labeled “conducive communities”). These included social and support groups [158-167], interventions centered around providing social services [168-171], education-based groups [172-175], and peer mentoring [176-178]. Most support groups were geared toward subpopulations, including older adult migrants, low-income or frail older adults, those with new or chronic disabilities, and those facing specific difficulties in community mobility. Many groups were also tailored specifically for men or women, such as men’s social groups [164] or support groups for older single women [174] or widows [161,167].

#### Promoting Mental and Physical Well-being

The second theme grouped interventions promoting mental and physical well-being through activities that support the adoption and maintenance of healthy living for older adults. These interventions included regular physical and recreational activities, often group based and offered at a community activity center and facilitated by trained instructors. Psychological therapy facilitated by trained professionals, such as psychologists, counselors, social workers, or nurses, was another common intervention [179-184]. These programs were offered in person, primarily in group settings at older adult centers on a weekly basis, and were either focused on a particular therapeutical approach (eg, reminiscence group therapy [182] or mindfulness-based stress reduction) or specific populations (eg, bereaved widows [179] or older adults living with mental health issues [183]). Interventions under this theme also included programs offering companionship and comfort with nonhuman companions, such as domestic or virtual animals or robopets.

#### Providing Instrumental Support

The third theme grouped interventions that offered instrumental support to older adults requiring an extra level of functional support, such as those with visual or hearing impairments, dementia, or other chronic disabilities, with the objective of enabling independent functioning and the capacity to engage with available social services. These interventions included personalized support and linkage to services provided by occupational therapists and allied health care professionals as well as the use of assistive technologies. OT interventions included programs engaging older adults to adjust their lifestyle and target the source of their social deficits, such as holistic lifestyle programs and wellness educational courses [185-201]. Assistive technology solutions aimed to provide older adults reliable daily support through user-friendly tools such as assistive robots to maintain or improve their quality of life, given cognitive or functional impairments [202], and to meet their social needs independently.

#### Providing Home and Community Care

Several interventions included at-home care services provided by a clinician, allied health professional, or support worker that directly or indirectly targeted social isolation and loneliness and facilitated healthy independent living for older adults. These services included home-based health services [203-207] as well as professional telehealth care provided through phone [208-212] or web-based [213-215] systems. These interventions primarily targeted the outcomes of social isolation and loneliness through one-on-one services. Some visits combined the screening with referral services [216-218].

### Effectiveness

**Figure 3:**
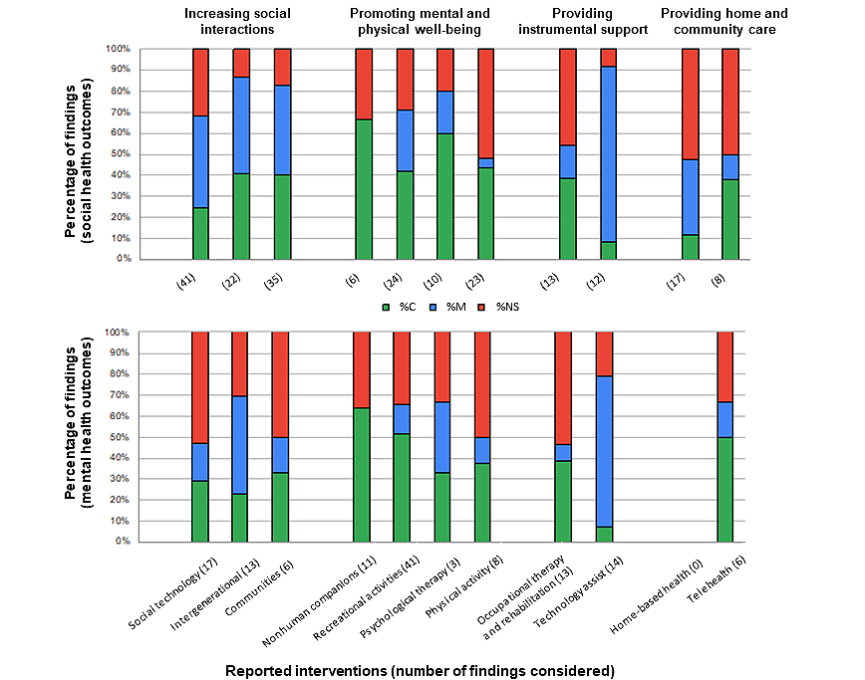
Percentage of findings related to social (top part of figure) and mental health (bottom part of figure) outcomes for each of the 11 intervention subcategories evaluated as either consistent (C; green), mixed (M; blue), or not supporting (NS; red) effectiveness (numbers in the parentheses represent the number of findings considered to calculate the effectiveness of each type of intervention).

Figure 3 presents the proportion of effective interventions (ie, convincing, mixed, or no evidence for effectiveness) per intervention category for each type of outcome (mental or social health). The number of individual findings used to calculate each effectiveness is also shown in the figure. Interventions falling under >1 intervention type were classified according to the primary focus of the intervention (eg, recreation-based support groups are categorized under recreational activity interventions as opposed to interventions focusing on conducive communities). As numerous primary studies were cited and described across several reviews concurrently, each paper was accounted for only once and classified based on the targeted outcomes of the intervention reported (ie, addressing social or mental health outcomes).

Collectively, interventions under the themes of increasing social interactions and promoting mental and physical well-being were the most effective at improving social outcomes, with a higher proportion of interventions demonstrating clear effectiveness. Interventions targeting the promotion of well-being also showed the greatest success for improving mental health outcomes. Interventions under the theme of improving social health through home and community care were also effective in improving mental health, although fewer findings were available for this type of intervention.

A total of 80 primary studies reported on the first theme: *increasing social interactions*. These interventions primarily targeted social health outcomes, including reducing loneliness and increasing social networks and social support, and their effectiveness was reported as primarily consistent or mixed. Interventions aimed at increasing social interactions that took place outside the home or were group based appeared to be more effective at improving social health outcomes (refer to the summary findings in Multimedia Appendix 3). ICT interventions (reported in 30 studies) were found to be associated with increased social networks and reduced feelings of loneliness. Group sessions were more effective than one-on-one ICT training sessions for decreasing loneliness and increasing the mental well-being of participants [137]. Conducive communities (reported in 22 studies), including social and support groups with structured discussions on relevant topics, increased participant self-confidence, increased involvement, and reduced social isolation [159]. Examples of topics included information on personal safety, nutrition, general wellness, successful aging, coping strategies, the management of existing relationships, and strategies for building new relationships. Finally, intergenerational interventions (reported in 28 studies) showed greater effectiveness (consistent and mixed) with respect to reducing mental health outcomes, such as symptoms of depression or anxiety, compared with conducive communities or social technology (refer to the study by Paquet et al [45]).

Interventions *promoting mental and physical well-being* were the focus of 80 primary studies. Among these, nonhuman companions (reported in 12 studies) were markedly effective in improving both mental and social health outcomes, primarily targeting depression, anxiety, and loneliness. Participants often received one-on-one contact with an animal [219-224] or robopet [225-230] through brief home visits or longer stays, demonstrating comparative effectiveness across companion types (ie, dogs, birds, crickets, aquarium animals, virtual pets, and robopets). Recreational activities (reported in 39 studies) were particularly effective in improving both mental and social outcomes. These primarily targeted mental well-being, depression, social isolation, and loneliness, with music-based interventions comprising a high percentage of the effective activities administered [231-242], followed closely by recreational activities with a social group focus [177,197,243-249]. Interventions that cultivate meaningful roles within a group were most effective [250]. Social groups involving leisure activities and interventions promoting autonomy by encouraging participants to select their own social activities were most effective in reducing social isolation and loneliness [177,247,248]. Cumulatively, physical activity interventions (n=23 studies) were effective in reducing loneliness and depression and increasing social networks and social support; however, outcomes appeared to fluctuate depending on the type of intervention administered. Physical activities such as exergames [251-257] and specialized exercise programs featuring a recreational or educational component were the most common and effective [168,258-261]. Specifically, studies that combined exercise with other activities provided greater benefits than those that offered exercise alone. Therapy-focused interventions (n=6 studies), such as reminiscence group therapy [182] or mindfulness-based stress reduction [180], were effective in reducing social isolation and loneliness. However, several studies reporting their effectiveness had small sample sizes, a high risk of bias, or were of poor quality [180-182].

Interventions under the *instrumental support* theme (n=39 studies) primarily targeted the mental well-being of older adults. OT interventions (n=23 studies) reported as effective primarily included holistic lifestyle programs and wellness educational courses [185-201]. For example, the Lifestyle Redesign program, an OT initiative encompassing education, discussions of personal experiences, and recreational activities was most effective in increasing social function and reducing depressive symptoms [187,195,196]. Most studies regarding assistive technology (n=16 studies) reported on assistive robots [262-270], such as the seal-like Paro robot, to facilitate social interactions and aid in daily living activities [263,266,267,269,270], and significant improvements in psychological well-being were reported during group interactions [271]. Small humanoid robots were particularly effective at improving social engagement and providing a trusting, emotional relationship [265]. Nevertheless, most assistive technology interventions provided mixed effectiveness and required further investigation as most studies were pilot studies or had small sample sizes and few were randomized control trials.

Finally, for interventions related to *home and community care* (n=19 studies), home-based health services (n=11 studies), which encompassed screening for social isolation, were presented as an effective means of improving social health outcomes, including reducing social isolation or loneliness [216,272,273]. Cattan et al [47] and Gardiner et al [48] reported that intervention programs incorporating inputs from the target population during the planning, development, and execution stages were also more likely to be successful. With respect to telehealth, interventions focused on reducing the symptoms of depression, loneliness, and social isolation (n=8 studies) and only a few interventions were found to be effective. These included an internet-based health maintenance platform for patients with diabetes [213], which successfully decreased depression and improved social support, and a suicide crisis phone line for older adults, which was found to be effective in reducing loneliness and partially effective in reducing social isolation [212].

Additional characteristics contributing to the effectiveness of interventions across themes were extracted from review-level conclusions. Most consistently, reviews reported on the success of group interventions involving educational and social support input [47]. In agreement, Dickens et al [49] and Papageorgiou et al [50] noted the greater effectiveness of activity-based or social support groups and interventions with an educational or skill development focus [50,51]. However, Gardiner et al [48] and Cohen-Mansfield and Perach [51] state that the activity or purpose behind group gatherings is likely to play a major part in the effectiveness of improving outcomes. Other observations included the importance of participatory methods in which older people are involved in planning, developing, and delivering activities [47] and the use of theory-informed [49] and multistrategy approaches [38]. Finally, several reviews expressed support for the use and development of technology for tackling issues of social isolation and loneliness in older adults [38,51-53].

### Comparing Terms From the Literature Versus Community Data Sources

Considering that some degree of effectiveness was observed across all intervention types, the intervention terms were derived for all categories to investigate their presence and visibility within the selected data sources. In addition to the reviews included in the meta-review, we considered the available published taxonomies for social isolation or loneliness interventions. Of the 3 studies identified [274-276], only the findings from the study by Leung et al [274] were of relevance, given their classification of late-life leisure activities for increasing social participation. An average of 28 (SD 13.4) terms per intervention category were identified from the literature (Table 2).

**Figure 4:**
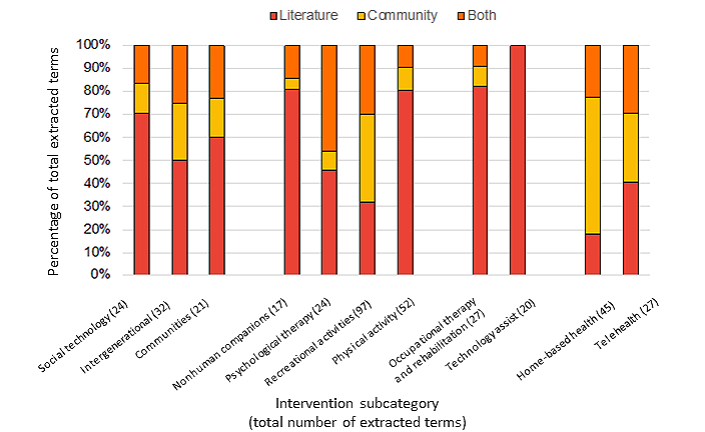
Percentage of keywords extracted from the literature-based resources only (red), community web-based data sources only (yellow), or both (orange), for each intervention subcategory.

For each intervention category, we extracted relevant terms and activities from the community data sources outlined in Table 1 and compared them with those derived from the literature. The objective was to characterize the nature and the proportion of terms that were either specific to the literature or the community sources or common across both (Figure 4; Table 2). Table 2 also reports the percentage of keywords extracted from each type of data source (ie, regional and municipal, neighborhood specific, and population specific). Interventions for which there was a stronger overlap (³30%) between the terms identified in the literature and services (and respective keywords) present in community data sources included psychological therapy, recreational activities, and telehealth. In contrast, there were relatively fewer terms related to ICT, assistive technology, OT services, and nonhuman companions in the selected community data sources, whereas a substantially larger proportion of terms related to physical activity interventions were extracted from the literature.

Most overlapping keywords that described primary health care interventions such as home-based health services and telehealth, occupational, and psychological therapies were extracted from regional data sources (ie, Données Montréal [100] or 211 Grand Montréal [99]). In contrast, interventions or services aimed at increasing social interactions (apart from intergenerational interventions) were offered primarily at the population level, that is, those specific to the older population within Montréal (eg, Contactivity Centre [102] and AMI-Québec [103]). This was consistent for interventions and services related to physical activity, whereas recreational activities were primarily offered at both the population and regional levels. “Conducive communities” was the only intervention subcategory that presented over half of the overlapping terms in sources at the neighborhood level (Table 2).

**Table 2:** Comparison of literature-based versus community-based intervention keywords or terms.

Intervention theme and subthemes				Terms present in										Data sources providing keywords (“Community Only” or “Both”)	
				Literature only			Community data sources only			Both					
**Increasing social interactions**															
		Social technology			ICT, training, internet, social, network*, computer, telephone, befriending, videocall, group, internet-based, activit*, telecommunicat*, teleconference*, videoconferenc*, radio, Ipad			tech*, program, workshop			Support, friend*, call*, phone				100% (7/7) population
		Intergenerational interventions			reminiscence, tutor*, mentor*, video, game, craft, physical activit*, retreat, model*, CARELINK, play*, volunteer*, life-skill, art*, music, talk*			workshop, listen*, support, book club, connect old* to young*, friend*, visit*			Intergeneration*, reading, sharing, discussion, educat*, story, recreation*, teach*				87% (13/15) regional, 7% (1/15) neighborhood, and 27% (4/15) population
		Conducive communities			mentor*, befriending, education, social service, self-help, self-management, buddy, leisure, companion, discussion, bereavement, volunteer*, club, lesson, meeting, connect*, cultural*, health, promotion, peer, exchange			visit*, friend*, meeting, meal*, wellness, lunch*			centre, friendship, communit*, program, support, network, group, social				29% (4/14) regional, 57% (8/14) neighborhood, and 86% (12/14) population
**Promoting mental and physical well-being**															
		Nonhuman companions			Robo-pet (IrobiQ, Cafero, AIBO, WOBOT, Gerijoy), animal, assist*, bird*, virtual, pet, companion, cricket*, care*, lifestyle, aquarium, robo*			Cat*			Therapy, dog*, visit*				100% (4/4) population
		Psychological therapy			writing, rehab*, reminiscence, self-help, art, discussion, social support, mindfulness, stress-reduc*, self-practice, sharing			Follow-up, advice			support, group, consult*, referral, meeting, active listen*, listen*, psychotherap*, therap*, counsel*, psychosocial				77% (10/13) regional, 38% (5/13) neighborhood, and 31% (4/13) population
		Recreational activities			Horticultur*, sing*, plant*, video, Wii, chorale, writing, paint*, day group, drama, group, reflect, choir, gender, meet*, friend*, outdoor, act*, solitair*, service, lay*, read*, forums, handicraft, fishing, shopping, facial, massage, concert, opera, religio*			Fit*, recreation*, literature, health*, education*, food, meal*, sport*, computer, lunch*, dinner, yoga, outing, library, cinema, auditorium, gallery, planetarium, insectarium, pool, mall, histor*, movie, market, ski, sound, nature, leisure, conference, socializ*, holiday, course*, trip, craft, entertainment, shows, hobb*			danc*, art, cultur*, music, class*, social, support, communit*, network, activit*, workshop, discussion, volunteer*, game, swim*, museum, exhibit*, perform*, theatre, centre, garden, park, walk*, cook*, picnic, program, club, bowling, bingo				76% (50/66) regional, 17% (11/66) neighborhood, and 52% (34/66) population
		Physical activity			Exergame*, Wii, fit, martial art, qigong, walk*, health*, train*, breath*, strength*, flexibil*, aerobic, education*, resistance, pool, nutrition*, soccer, ski, at-home, group, tennis, bowling, baseball, boxing, nonaerobic, ton* (tone/toning), self-care, discussion, promotion, gym, balance, table tennis, golf, cycl*, Mind-body, jog*, run*, hiking, ballgame, calisthenics, danc*, stretch*			wellbeing, activit*, outdoor, viactive, centre			Physical, exercise, tai chi, yoga, sport*				40% (4/10) neighborhood and 80% (8/10) population
**Providing instrumental support**															
		Occupational therapy and rehabilitation			Psychosocial, lifestyle, occupation*, self-management, discussion, education*, treatment plan, goal*, activit*, physical, train*, peer, shar*, exchange, assistive, device, client-centered, health promotion, recreation*, individualized, mobility, cognition, mood, behavior, nutrition, support, home			Direct intervention, consult*, health*			Group, therap*, program				100% (6/6) regional
		Assistive technology			Assist*, robot*, sensory, tech*, smart, phone, watch, system, PARO, aid, humanoid, mobile, remote, multimedia, tablet, cloud, personalized, communication, caregiver, TV			—^a^			—				—
**Providing home and community care**															
	Home-based health services		screening, hospital-to-home, social, profession*, at-risk, needs, resource*, discharge			Caregiver, care, group, conference, information, transition (following hospital care), frontline, intervention, follow-up, long-term, check-up, emergency, security, hous*, accompan*, drop-in, respite, promotion, home, listen*, guid*, home care, transport*, education, news, service directory, fall prevention class			At-home, visit*, home, support, referral, service, outreach, assist*, health, hospital			65% regional (24/37), 38% (14/37) neighborhood, and 24% (9/37) population			
	Telehealth		Internet-based, maintain*, problem-solv*, goal*, web-based, telesupport, crisis, track*, educat*, computer, conference			referral, check-up, medication reminder call, consult*, active listen*, listen*, psychotherapy*			call*, telephone, phone, support, group, counsel*, therap*, health			100% (15/15) regional and 33% (5/15) population			

^a^None of the included community sources reported relevant keywords that reflect assistive technology.

## Discussion

### Principal Findings

This study identified and assessed the effectiveness of interventions designed to reduce social isolation and loneliness in older adults through a meta-review. Derived from this literature, it then compared key terms with those extracted from directories of municipal and community data sources serving the population of Montréal, a city in which over one-third of older adults live alone and two-thirds of those living with a disability report difficulties in participating in social activities within their community [98]. These realities translate to significant strain on the health care system given the close association of social isolation with, for example, depression, dementia, poor general health, caregiver burden, and increased falls [277].

Key terms were further classified according to four intervention types emerging from the meta-review: (1) increasing social interactions, (2) promoting mental and physical well-being, (3) providing instrumental support, and (4) improving social health through home and community care, and each of these presented a unique strategy for improving social isolation and loneliness and mitigating the impact on well-being, resilience, and coping in older adults.

For interventions aimed at promoting mental and physical well-being, many key terms from the literature review related to these interventions overlapped with those extracted from web-based community data sources, specifically in the areas of recreational activities and psychological therapies. Overall, however, the process of comparing extracted intervention terms across literature and community-based data sources indicated an overlap in less than half of the total keywords within each category, emphasizing the need for greater consistency. A shared or common nomenclature for navigating community-based interventions would assist in identifying potential gaps in local services and in tracking the effectiveness of the current on-the-ground interventions. These functions can inform the development and sustainability of social prescription programs [42] that successfully address the social needs of older persons (refer to the reviews by Chatterjee et al [278] and Bickerdike et al [279]).

### “Effective” Interventions for Reducing Social Isolation and Loneliness

The bulk of interventions reviewed in this study fell in the areas of increasing social interactions or promoting mental and physical well-being, where the former predominantly targeted social health outcomes. Intergenerational interventions and “conducive communities” interventions presented >70% consistent or mixed effectiveness for outcomes such as reducing loneliness and increasing social networks and social support. Comparably, psychological therapy and recreational activity interventions demonstrated positive outcomes for reducing loneliness and targeting mental health outcomes (>60% consistent or mixed), such as depression and mental well-being. Although fewer interventions reported on mental health outcomes, nonhuman companions and telehealth subcategories were effective (>60%) in improving mental health outcomes, particularly depression and anxiety.

Collectively, interventions were more successful when participants took a meaningful role within the group [250], when a collective purpose was evident [48,51], when participants experienced autonomy [246], and when participants contributed to the development of the intervention [47]. Participants also found value in interventions with an educational component [47] where new skills and insights could be integrated into their day-to-day living [172], as well as in interventions that implemented multiple strategies [38]. Many successful interventions incorporated some technology integration, such as social cognitive training via telehealth, enabling social interactions through training and access to ICT, improving social support through online support groups, or accessing social robots and virtual pets. Digital infrastructure, along with other comparable smart technologies, has been shown to support aging, allowing individuals with disability to maintain a more social life. Digital interventions in social care have become increasingly valued and used on the heels of COVID-19, providing relief for the isolated and those living in remote areas. Nonetheless, improved digital literacy, greater economic and occupational access to technology, and measures to ensure privacy and security are crucial for enabling the uptake of digital interventions among older adults [280].

The study findings also emphasized the value of personalized services based on an assessment of patient social needs and risk factors. For example, environmental or situational factors, such as living alone; limited access to social support or social networks [33,170,281-311]; cognitive, mental or physical deficits (eg, chronic illness, visual or hearing impairments, and depression); and other demographic and socioeconomic attributes such as age, gender, marital status, immigration status, education, and income [33,170,286,289,292,294,298,299,302,304-306,308-310,312-375] were more likely to influence or be associated with loneliness [45]. Recognizing the diversity of needs and preferences of older populations and the importance of user participation in the development of interventions to address social isolation and loneliness, it is likely that tailored approaches will be more successful than “one-size-fits-all” solutions. Each of the 11 intervention categories may preferentially benefit one or more of these unique populations. For example, older adults with greater mobility may be more inclined to take part in recreational or physical activity interventions, whereas those requiring greater physical support may derive more benefit from home-based health services or OT and rehabilitation. Individual preferences and characteristics can be considered during the social prescription process through the use of a digital platform that integrates patient information captured in medical records with information on available services, their nature, and their effectiveness or relevance for different subgroups of the population. This path toward personalized care and patient engagement in care decision-making presents an opportunity to improve social health outcomes, reduce costs, and improve overall patient satisfaction [376].

### Bridging the Gap Between Scientific and Community-Produced Evidence

In building decision tools that facilitate the delivery of personalized nonmedical care, a thorough inventory of interventions currently accessible to the community is needed to identify service gaps and assess the quality of services. A cross-comparison of intervention keywords extracted from web-based community databases in Montréal versus literature-based keywords demonstrated an overlap of nearly 50% for psychological therapies and substantial overlap (≥25%) for recreational activities, telehealth, and intergenerational interventions. Notably, many of these terms were extracted from regional sources, such as the 211 web-based directory [99], where each organization’s file includes details of services and programs offered, the targeted user, eligibility criteria, etc.

Despite the observed overlap, some discrepancies were also observed, with terms related to certain types of interventions being overrepresented, either in the literature or in community resources. For instance, little evidence of interventions (and key terms) related to physical activity, OT, assistive technologies, nonhuman companions, or social technology was observed in the community databases. Many of these less accessible interventions are rooted in physical health and may not necessarily be covered by the sources examined. Moreover, some of these interventions incorporate some aspect of technology, which is a relatively novel means for addressing social isolation and loneliness, with related implementation challenges including cost or feasibility of adoption. In contrast, home-based health service terms were disproportionally fewer in the literature as compared with community services, likely reflecting the nature of our search strategy, where excluding reviews focused on institutional settings may have reduced the terminology related to the transition of care from hospital to home. However, these strategies have received more attention during the COVID-19 pandemic [280,377]. It should be noted, however, that our search did not investigate health care–specific community resources. Finally, in view of the inconsistent overlap between community data sources offered at the neighborhood level and the literature, investment in categorizing and advertising community services is needed and keeping service data up-to-date. The lack of up-to-date contact and service information has contributed to health care providers’ reticence in making patient referrals to local community sources [378]. This information includes whether the services are available, financially accessible, and proximate to those in need. In general, data sources varied substantially in terms of the level of detail provided regarding service description, emphasizing the need for greater consistency in how information is captured [99]. Such information would also help support the activities of link workers who play a critical role in social prescription programs, offering service referrals and ongoing support to users as well as collecting data for intervention evaluation and analyses [40].

### Road Map to a Common Language

Developing a social prescription ontology provides the framework for a clinical decision aid that could lessen the burden of link workers and provide increasing value to those in need. An ontology provides a means of sharing machine-interpretable domain information across stakeholders, whereby information is defined into concepts (also referred to as classes or entities) and attributes (ie, slot, roles, or properties) of said classes, and the relationships among them are specified [379,380]. The W3C Web Ontology Language is a computational logic-based language used to represent an ontology. The W3C Web Ontology Language formally structures and describes the semantics of ontology terms, such that computers can feasibly conduct reasoning tasks on ontologies and databases that use them [381]. The Open Biological and Biomedical Ontology Foundry is another initiative aiming to develop a family of interoperable ontologies that is logically organized and scientifically accurate [382]. A key principle underlying these efforts is that ontologies be findable, accessible, reusable, and interoperable and consist of content that is both scientifically sound and meets community needs [383,384]. Of similar importance is accurate categorization for data acquisition, analysis, and interpretation [274] and the successful implementation and evaluation of interventions to reduce social isolation and loneliness in older adults. Accordingly, ontology development should engage domain experts to promote its accurate and impactful incorporation into practice [380,385]. For social prescription, this includes engaging a full spectrum of users in the development process so that the resultant ontologies enable and reflect their navigation and decision support needs [386]. Although efforts are underway to standardize information related to social needs from a clinical informatics perspective, similar efforts to organize knowledge about the resources required to meet these needs are still lagging. Given the range of multisectoral users requiring decision support for social prescription, user-centered design processes must engage input from not only health professionals but also community organizations, public health services, and older adult community partners and caregivers [387]. The involvement of users in ontology and decision tool development will also promote their use [388], deepen community ties [389], and encourage support and participation from those responsible for funding and promoting services within the community [390].

Likewise, it is important that decision support resources are comprehensive and intelligible and categorized using simple terms, given that a large proportion of users (directly or indirectly) will be older adults, often presenting comorbidities such as visual, hearing, and mild cognitive impairments [274]. The language used must be formal, shared, and broadly applicable [391] and have a structure that can be easily adopted by those designing future interventions [274]. With respect to the social prescription project, further development will involve extending, specializing, adapting, and unifying ontologies or taxonomies by merging multiple sets in a similar subject matter [392] to eliminate duplication [386,393]. The 211 database [99], a taxonomy of human needs covering a range of community services, provides a promising basis for this work. The content, design, and functionality of the decision support tool developed will be refined over time to improve performance or respond to needs as they evolve. The digital platform can, in real time, process data for service users, link workers, and health care professionals to monitor and manage use; measure outcomes; evaluate interventions; and integrate insights from experts in the field [388].

### Limitations and Future Directions

Implicit in the use of existing reviews as a main source of information for the knowledge synthesis are uncertainties about the quality of the search strategies used and the level of details on primary studies reported in the individual reviews. Moreover, owing to time limitations, a search was not conducted to capture primary papers not covered by the reviewed studies, with the most recent review year being 2018. To address this limitation, we used an adapted quality appraisal tool to identify the shortcomings of these reviews. It is important to note that many of the studies did not report on effectiveness, and they did not meet the quality appraisal criteria related to duplication of data extractions or consideration for risk of bias. Finally, as the number of outcomes varied across intervention subcategories, further analyses will be required to evaluate interventions with limited evidence. Once developed, the digital tool has the potential to provide better metrics for assessing such programs and integrating these assessments to improve performance. Furthermore, there is considerable potential for data mining, artificial intelligence, and data science techniques to not only prevent the occurrence of inaccurate, out-of-date, or missing information, thereby improving the profiling and clustering of users, but also predict patients’ service preferences. Such a tool could include features that permit older adult needs assessments and tracking, incorporate social prescription referrals into primary care practice, and enable personalized matching of patient needs and locally available resources. Evergreen Life [17] and Elemental [18] are 2 early examples of software used in the United Kingdom to pair needs assessed from electronic patient records with nonmedical services offered within community directories.

### Conclusions

Our findings demonstrate that a number of interventions have been shown to be effective at improving mental and social health outcomes of socially isolated or lonely older adults and that many of these interventions are already available services to the Montréal population, although terms to refer to such interventions or services might differ between the literature and the information sources provided to the community. Our findings also support the value and opportunity of developing a knowledge structure around interventions and resources to meet the identified social needs of individuals as part of an efficient social prescription ontology that describes all core processes of social prescription. These include the assessment of needs, participation in relevant community activities meeting those needs, and the outcomes of participation. This structure would also help in identifying and compiling relevant resources. As service gaps remain unaddressed, these tools and strategies are urgently required to address the changing social needs of older adults and support aging in place. Efforts to harmonize data sources and accurately identifying services that address the specific social needs of vulnerable individuals will inform ongoing efforts to develop digitally based social prescription tools to address SDOH, without further taxing an already overburdened health care system.

## Appendix

Multimedia Appendix 1 Search terms.

Multimedia Appendix 2 List of reviews included with types of interventions reviewed.

Multimedia Appendix 3 Interventions and effectiveness.

## Abbreviations

AMSTAR: A Measurement Tool to Assess Systematic Reviews
EMIS: Egton Medical Information Systems
FADOQ: Réseau Fédération de l’âge d’or du Québec
ICT: information and communication technology
OT: occupational therapy
PRISMA: Preferred Reporting Items for Systematic Reviews and Meta-Analyses
RIAQ: Réseau d’information des aînés du Québec
SDOH: social determinants of health

## References

[1] DeVoe J, Bazemore AW, Cottrell EK, Likumahuwa-Ackman S, Grandmont J, Spach N, Gold R (2016). Perspectives in primary care: a conceptual framework and path for integrating social determinants of health into primary care practice. Ann Fam Med.

[2] Pilkington K, Loef M, Polley M (2017). Searching for real-world effectiveness of health care innovations: scoping study of social prescribing for diabetes. J Med Internet Res.

[3] Polley M, Pilkington K (2017). A review of the evidence assessing impact of social prescribing on healthcare demand and cost implications. University of Westminster.

[4] Nowak DA, Mulligan K (2021). Social prescribing: a call to action. Can Fam Physician.

[5] Andermann A, CLEAR Collaboration (2016). Taking action on the social determinants of health in clinical practice: a framework for health professionals. CMAJ.

[6] Gurewich D, Garg A, Kressin NR (2020). Addressing social determinants of health within healthcare delivery systems: a framework to ground and inform health outcomes. J Gen Intern Med.

[7] Marmot M (2017). Social justice, epidemiology and health inequalities. Eur J Epidemiol.

[8] Wilensky G (2016). Addressing social issues affecting health to improve US health outcomes. JAMA.

[9] Bradley EH, Canavan M, Rogan E, Talbert-Slagle K, Ndumele C, Taylor L, Curry LA (2016). Variation in health outcomes: the role of spending on social services, public health, and health care, 2000-09. Health Aff (Millwood).

[10] Peretz PJ, Islam N, Matiz LA (2020). Community health workers and covid-19 — addressing social determinants of health in times of crisis and beyond. N Engl J Med.

[11] Dubuc N, Brière S, Corbin C, N'Bouke A, Bonin L, Delli-Colli N (2021). Computerized care-pathways (CCPs) system to support person-centered, integrated, and proactive care in home-care settings. Inform Health Soc Care.

[12] Brandling J, House W (2009). Social prescribing in general practice: adding meaning to medicine. Br J Gen Pract.

[13] Drinkwater C, Wildman J, Moffatt S (2019). Social prescribing. BMJ.

[14] Jungmann S, Mistry P, Conibear T, Gray M, Jani A (2020). Using technology-enabled social prescriptions to disrupt healthcare. J R Soc Med.

[15] Skivington K, Smith M, Chng NR, Mackenzie M, Wyke S, Mercer SW (2018). Delivering a primary care-based social prescribing initiative: a qualitative study of the benefits and challenges. Br J Gen Pract.

[16] Polley M, Fleming J, Anfilogoff T, Carpenter A (2017). Making sense of social prescribing. University of Westminster.

[17] Evergreen Life homepage. Evergreen Life.

[18] Elemental social prescription connector. EMIS Health.

[19] Hollander AD, Hoy C, Huber PR, Hyder A, Lange MC, Latham A, Quinn JF, Riggle CM, Tomich TP (2019). Toward smart foodsheds: using stakeholder engagement to improve informatics frameworks for regional food systems. Annals Am Assoc Geographers.

[20] Harrow I, Balakrishnan R, Jimenez-Ruiz E, Jupp S, Lomax J, Reed J, Romacker M, Senger C, Splendiani A, Wilson J, Woollard P (2019). Ontology mapping for semantically enabled applications. Drug Discov Today.

[21] Rathore AS, Garcia-Aponte OF, Golabgir A, Vallejo-Diaz BM, Herwig C (2017). Role of knowledge management in development and lifecycle management of biopharmaceuticals. Pharm Res.

[22] Marques MM, Carey RN, Norris E, Evans F, Finnerty AN, Hastings J, Jenkins E, Johnston M, West R, Michie S (2020). Delivering behaviour change interventions: development of a mode of delivery ontology. Wellcome Open Res.

[23] Taye MM (2023). Understanding semantic web and ontologies: theory and applications. arXiv. Preprint posted online on June 23, 2010.

[24] (2023). Social prescribing information standard. Professional Records Standard Body.

[25] (2020). A commitment to grow social determinants of health content within SNOMED CT. SNOMED International.

[26] Kim H, Jung J (2021). Social isolation and psychological distress during the COVID-19 pandemic: a cross-national analysis. Gerontologist.

[27] Holmes EA, O'Connor RC, Perry VH, Tracey I, Wessely S, Arseneault L, Ballard C, Christensen H, Cohen Silver R, Everall I, Ford T, John A, Kabir T, King K, Madan I, Michie S, Przybylski AK, Shafran R, Sweeney A, Worthman CM, Yardley L, Cowan K, Cope C, Hotopf M, Bullmore E (2020). Multidisciplinary research priorities for the COVID-19 pandemic: a call for action for mental health science. Lancet Psychiatry.

[28] Courtin E, Knapp M (2017). Social isolation, loneliness and health in old age: a scoping review. Health Soc Care Community.

[29] Santini ZI, Jose PE, York Cornwell E, Koyanagi A, Nielsen L, Hinrichsen C, Meilstrup C, Madsen KR, Koushede V (2020). Social disconnectedness, perceived isolation, and symptoms of depression and anxiety among older Americans (NSHAP): a longitudinal mediation analysis. Lancet Public Health.

[30] Evans IE, Llewellyn DJ, Matthews FE, Woods RT, Brayne C, Clare L, CFAS-Wales research team (2018). Social isolation, cognitive reserve, and cognition in healthy older people. PLoS One.

[31] Evans IE, Martyr A, Collins R, Brayne C, Clare L (2019). Social isolation and cognitive function in later life: a systematic review and meta-analysis. J Alzheimer's Disease.

[32] Lara E, Martín-María N, De la Torre-Luque A, Koyanagi A, Vancampfort D, Izquierdo A, Miret M (2019). Does loneliness contribute to mild cognitive impairment and dementia? A systematic review and meta-analysis of longitudinal studies. Ageing Res Rev.

[33] Perissinotto CM, Stijacic Cenzer I, Covinsky KE (2012). Loneliness in older persons: a predictor of functional decline and death. Arch Intern Med.

[34] Valtorta NK, Kanaan M, Gilbody S, Hanratty B (2018). Loneliness, social isolation and risk of cardiovascular disease in the English Longitudinal Study of Ageing. Eur J Prev Cardiol.

[35] Elovainio M, Hakulinen C, Pulkki-Råback L, Virtanen M, Josefsson K, Jokela M, Vahtera J, Kivimäki M (2017). Contribution of risk factors to excess mortality in isolated and lonely individuals: an analysis of data from the UK Biobank cohort study. Lancet Public Health.

[36] Pantell M, Rehkopf D, Jutte D, Syme SL, Balmes J, Adler N (2013). Social isolation: a predictor of mortality comparable to traditional clinical risk factors. Am J Public Health.

[37] (2019). World Population Prospects 2019: Highlights. United Nations Department of Economic and Social Affairs.

[38] Poscia A, Stojanovic J, La Milia DI, Duplaga M, Grysztar M, Moscato U, Onder G, Collamati A, Ricciardi W, Magnavita N (2018). Interventions targeting loneliness and social isolation among the older people: an update systematic review. Exp Gerontol.

[39] Baker E, Clark LL (2020). Biopsychopharmacosocial approach to assess impact of social distancing and isolation on mental health in older adults. Br J Community Nurs.

[40] Pescheny J, Randhawa G, Pappas Y (2020). The impact of social prescribing services on service users: a systematic review of the evidence. Eur J Public Health.

[41] Carlile PR (2004). Transferring, translating, and transforming: an integrative framework for managing knowledge across boundaries. Organization Sci.

[42] Jani A, Liyanage H, Okusi C, Sherlock J, Hoang U, Ferreira F, Yonova I, de Lusignan S (2020). Using an ontology to facilitate more accurate coding of social prescriptions addressing social determinants of health: feasibility study. J Med Internet Res.

[43] Moher D, Liberati A, Tetzlaff J, Altman DG, PRISMA Group (2009). Preferred reporting items for systematic reviews and meta-analyses: the PRISMA statement. PLoS Med.

[44] Xiao Y, Watson M (2017). Guidance on conducting a systematic literature review. J Planning Educ Res.

[45] Paquet C, Whitehead JC, Cisneros-Franco JM, Adams AM, Shah R, Sengupta R, Aunio A-L, Moore S, D’Aoust T, Kamar J, Zamorano T, Towell K, Um T, Issa AM, Couturier Y, Kaiser D, Dubé L (2020). Social isolation and loneliness in older adults during the COVID-19 pandemic: a knowledge synthesis of pre- and post-COVID 19 interventions and vulnerability and resilience factors. COVID 19 Mental Health Research.

[46] Shea BJ, Grimshaw JM, Wells GA, Boers M, Andersson N, Hamel C, Porter AC, Tugwell P, Moher D, Bouter LM (2007). Development of AMSTAR: a measurement tool to assess the methodological quality of systematic reviews. BMC Med Res Methodol.

[47] Cattan M, White M, Bond J, Learmouth A (2005). Preventing social isolation and loneliness among older people: a systematic review of health promotion interventions. Ageing Soc.

[48] Gardiner C, Geldenhuys G, Gott M (2018). Interventions to reduce social isolation and loneliness among older people: an integrative review. Health Soc Care Community.

[49] Dickens AP, Richards SH, Greaves CJ, Campbell JL (2011). Interventions targeting social isolation in older people: a systematic review. BMC Public Health.

[50] Papageorgiou N, Marquis R, Dare J, Batten R (2016). Occupational therapy and occupational participation in community dwelling older adults: a review of the evidence. Physical Occupational Therapy Geriatrics.

[51] Cohen-Mansfield J, Perach R (2015). Interventions for alleviating loneliness among older persons: a critical review. Am J Health Promot.

[52] Chen YR, Schulz PJ (2016). The effect of information communication technology interventions on reducing social isolation in the elderly: a systematic review. J Med Internet Res.

[53] Choi M, Kong S, Jung D (2012). Computer and internet interventions for loneliness and depression in older adults: a meta-analysis. Healthc Inform Res.

[54] Abdi J, Al-Hindawi A, Ng T, Vizcaychipi MP (2018). Scoping review on the use of socially assistive robot technology in elderly care. BMJ Open.

[55] Bemelmans R, Gelderblom GJ, Jonker P, de Witte L (2012). Socially assistive robots in elderly care: a systematic review into effects and effectiveness. J Am Med Dir Assoc.

[56] Buyl R, Beogo I, Fobelets M, Deletroz C, Van Landuyt P, Dequanter S, Gorus E, Bourbonnais A, Giguère A, Lechasseur K, Gagnon M (2020). e-Health interventions for healthy aging: a systematic review. Syst Rev.

[57] Pedrozo Campos Antunes T, Souza Bulle de Oliveira A, Hudec R, Brusque Crocetta T, Ferreira de Lima Antão JY, de Almeida Barbosa RT, Guarnieri R, Massetti T, Garner DM, de Abreu LC (2019). Assistive technology for communication of older adults: a systematic review. Aging Ment Health.

[58] Chao YY, Scherer YK, Montgomery CA (2015). Effects of using Nintendo Wii™ exergames in older adults: a review of the literature. J Aging Health.

[59] Chen SC, Jones C, Moyle W (2018). Social robots for depression in older adults: a systematic review. J Nurs Scholarsh.

[60] Chipps J, Jarvis MA, Ramlall S (2017). The effectiveness of e-Interventions on reducing social isolation in older persons: a systematic review of systematic reviews. J Telemed Telecare.

[61] Forsman AK, Nordmyr J, Wahlbeck K (2011). Psychosocial interventions for the promotion of mental health and the prevention of depression among older adults. Health Promot Int.

[62] Forsman AK, Nordmyr J, Matosevic T, Park AL, Wahlbeck K, McDaid D (2018). Promoting mental wellbeing among older people: technology-based interventions. Health Promot Int.

[63] Franck L, Molyneux N, Parkinson L (2016). Systematic review of interventions addressing social isolation and depression in aged care clients. Qual Life Res.

[64] Galbraith B, Larkin H, Moorhouse A, Oomen T (2015). Intergenerational programs for persons with dementia: a scoping review. J Gerontol Soc Work.

[65] Gee NR, Mueller MK (2019). A systematic review of research on pet ownership and animal interactions among older adults. Anthrozoös.

[66] Gerritzen EV, Hull MJ, Verbeek H, Smith AE, de Boer B (2019). Successful elements of intergenerational dementia programs: a scoping review. J Intergenerational Relationship.

[67] Gualano MR, Voglino G, Bert F, Thomas R, Camussi E, Siliquini R (2017). The impact of intergenerational programs on children and older adults: a review. Int Psychogeriatr.

[68] Hagan R, Manktelow R, Taylor BJ, Mallett J (2014). Reducing loneliness amongst older people: a systematic search and narrative review. Aging Ment Health.

[69] Heaven B, Brown LJ, White M, Errington L, Mathers JC, Moffatt S (2013). Supporting well-being in retirement through meaningful social roles: systematic review of intervention studies. Milbank Q.

[70] Jarvis MA, Padmanabhanunni A, Balakrishna Y, Chipps J (2020). The effectiveness of interventions addressing loneliness in older persons: an umbrella review. Int J Africa Nurs Sci.

[71] Kachouie R, Sedighadeli S, Khosla R, Chu M (2014). Socially assistive robots in elderly care: a mixed-method systematic literature review. Int J Hum Comput Interact.

[72] Khosravi P, Ghapanchi AH (2016). Investigating the effectiveness of technologies applied to assist seniors: a systematic literature review. Int J Med Inform.

[73] Knight T, Skouteris H, Townsend M, Hooley M (2014). The act of giving: a systematic review of nonfamilial intergenerational interaction. J Intergenerational Relationships.

[74] Koo BM, Vizer LM (2019). Examining mobile technologies to support older adults with dementia through the lens of personhood and human needs: scoping review. JMIR Mhealth Uhealth.

[75] Lee K, Jarrott SE, Juckett LA (2019). Documented outcomes for older adults in intergenerational programming: a scoping review. J Intergenerational Relationships.

[76] Li J, Erdt M, Chen L, Cao Y, Lee S, Theng Y (2018). The social effects of exergames on older adults: systematic review and metric analysis. J Med Internet Res.

[77] Masi CM, Chen H, Hawkley LC, Cacioppo JT (2011). A meta-analysis of interventions to reduce loneliness. Pers Soc Psychol Rev.

[78] Medical Advisory Secretariat (2008). Social isolation in community-dwelling seniors: an evidence-based analysis. Ont Health Technol Assess Ser.

[79] Morris ME, Adair B, Ozanne E, Kurowski W, Miller KJ, Pearce AJ, Santamaria N, Long M, Ventura C, Said CM (2014). Smart technologies to enhance social connectedness in older people who live at home. Australas J Ageing.

[80] Nicholas SO, Giang AT, Yap PL (2019). The effectiveness of horticultural therapy on older adults: a systematic review. J Am Med Dir Assoc.

[81] Noice T, Noice H, Kramer A (2014). Participatory arts for older adults: a review of benefits and challenges. Gerontologist.

[82] Pool MS, Agyemang CO, Smalbrugge M (2017). Interventions to improve social determinants of health among elderly ethnic minority groups: a review. Eur J Public Health.

[83] Portz JD (2017). A review of web-based chronic disease self-management for older adults. Gerontechnology.

[84] Pu L, Moyle W, Jones C, Todorovic M (2019). The effectiveness of social robots for older adults: a systematic review and meta-analysis of randomized controlled studies. Gerontologist.

[85] Roets-Merken LM, Draskovic I, Zuidema SU, van Erp WS, Graff MJ, Kempen GI, Vernooij-Dassen MJ (2015). Effectiveness of rehabilitation interventions in improving emotional and functional status in hearing or visually impaired older adults: a systematic review with meta-analyses. Clin Rehabil.

[86] Sadarangani TR, Murali KP (2018). Service use, participation, experiences, and outcomes among older adult immigrants in American adult day service centers: an integrative review of the literature. Res Gerontol Nurs.

[87] Shishehgar M, Kerr D, Blake J (2018). A systematic review of research into how robotic technology can help older people. Smart Health.

[88] Shvedko A, Whittaker AC, Thompson JL, Greig CA (2018). Physical activity interventions for treatment of social isolation, loneliness or low social support in older adults: a systematic review and meta-analysis of randomised controlled trials. Psychol Sport Exercise.

[89] Sims-Gould J, Tong CE, Wallis-Mayer L, Ashe MC (2017). Reablement, reactivation, rehabilitation and restorative interventions with older adults in receipt of home care: a systematic review. J Am Med Dir Assoc.

[90] Smallfield S, Molitor WL (2018). Occupational therapy interventions supporting social participation and leisure engagement for community-dwelling older adults: a systematic review. Am J Occup Ther.

[91] Turcotte PL, Carrier A, Roy V, Levasseur M (2018). Occupational therapists' contributions to fostering older adults' social participation: a scoping review. British J Occup Ther.

[92] Veazie S, Gilbert J, Winchell K, Paynter R, Guise J (2019). Addressing social isolation to improve the health of older adults: a rapid review. Rapid Evidence Product.

[93] Virués-Ortega J, Pastor-Barriuso R, Castellote JM, Población A, de Pedro-Cuesta J (2011). Effect of animal-assisted therapy on the psychological and functional status of elderly populations and patients with psychiatric disorders: a meta-analysis. Health Psychol Rev.

[94] Wang D, MacMillan T (2013). The benefits of gardening for older adults: a systematic review of the literature. Activities Adaptation Aging.

[95] Zhang F, Kaufman D (2016). A review of intergenerational play for facilitating interactions and learning. Gerontechnology.

[96] Côte-des-Neiges–Notre-Dame-de-Grâce. Ville de Montreal.

[97] (2022). Social prescription. CIUSSS West-Central Montreal.

[98] (2017). Portrait des aînés de l'île de Montréal. Santé Montréal.

[99] (2018). Montreal Island Directory of Community Services. 211 Grand Montreal.

[100] Données Montréal. Montréal.

[101] Centraide of Greater Montreal homepage. Centraide of Greater Montreal.

[102] Contactivity Centre homepage. Contactivity Centre.

[103] Ami Quebéc: action on mental illness homepage. Ami Quebéc Action on mental illness.

[104] Réseau FADOQ. FADOQ.

[105] RIAQ homepage. RIAQ.

[106] Chua PH, Jung Y, Lwin MO, Theng Y (2013). Let’s play together: effects of video-game play on intergenerational perceptions among youth and elderly participants. Comput Human Behav.

[107] Hernandez CR, Gonzalez MZ (2008). Effects of intergenerational interaction on aging. Educational Gerontol.

[108] Jarrott SE, Bruno K (2003). Intergenerational activities involving persons with dementia: an observational assessment. Am J Alzheimers Dis Other Demen.

[109] Jones ED, Herrick C, York RF (2004). An intergenerational group benefits both emotionally disturbed youth and older adults. Issues Ment Health Nurs.

[110] Short‐DeGraff MA, Diamond K (1996). Intergenerational program effects on social responses of elderly adult day care members. Educational Gerontol.

[111] Teater B (2016). Intergenerational programs to promote active aging: the experiences and perspectives of older adults. Activities Adaptation Aging.

[112] Kamei T, Itoi W, Kajii F, Kawakami C, Hasegawa M, Sugimoto T (2011). Six month outcomes of an innovative weekly intergenerational day program with older adults and school-aged children in a Japanese urban community. Jpn J Nurs Sci.

[113] Newman S, Ward C (1992). An observational study of intergenerational activities and behavior change in dementing elders at adult day care centers. Int J Aging Hum Dev.

[114] Rice M, Cheong Y, Ng J, Chua P, Theng Y-L (2012). Co-creating games through intergenerational design workshops. Proceedings of the Designing Interactive Systems Conference.

[115] O'Rourke KA (1999). Intergenerational Programming: Yesterday's Memories, Today's Moments, and Tomorrow's Hopes.

[116] Portman TAA, Bartlett JR, Carlson LA (2010). Relational theory and intergenerational connectedness: a qualitative study. Adultspan J.

[117] Au A, Ng E, Garner B, Lai S, Chan K (2015). Proactive aging and intergenerational mentoring program to promote the well-being of older adults: pilot studies. Clin Gerontologist.

[118] Carlson MC, Saczynski JS, Rebok GW, Seeman T, Glass T, McGill S, Tielsch J, Frick KD, Hill J, Fried LP (2008). Exploring the effects of an "everyday" activity program on executive function and memory in older adults: experience Corps. Gerontologist.

[119] Frick KD, Carlson MC, Glass TA, McGill S, Rebok GW, Simpson C, Fried LP (2004). Modeled cost-effectiveness of the Experience Corps Baltimore based on a pilot randomized trial. J Urban Health.

[120] Herrmann DS, Sipsas-Herrmann A, Stafford M, Herrmann NC (2005). Benefits and risks of intergenerational program participation by senior citizens. Educational Gerontol.

[121] Tan EJ, Xue QL, Li T, Carlson MC, Fried LP (2006). Volunteering: a physical activity intervention for older adults--The Experience Corps program in Baltimore. J Urban Health.

[122] Fujiwara Y, Nishi M, Watanabe N, Lee S, Inoue K, Yoshida H, Sakuma N, Kureta Y, Ishii K, Uchida H, Kakuno F, Shinkai S (2006). An intergenerational health promotion program involving older adults in urban areas. "Research of Productivity by Intergenerational Sympathy (REPRINTS)": first-year experience and short-term effects. Article in Japanese. Nihon Koshu Eisei Zasshi.

[123] Fujiwara Y, Sakuma N, Ohba H, Nishi M, Lee S, Watanabe N, Kousa Y, Yoshida H, Fukaya T, Yajima S, Amano H, Kureta Y, Ishii K, Uchida H, Shinkai S (2009). REPRINTS: effects of an intergenerational health promotion program for older adults in Japan. J Intergenerational Relationship.

[124] Lohman H, Griffiths Y, Coppard BM, Cota L (2003). The power of book discussion groups in intergenerational learning. Educational Gerontol.

[125] Murayama Y, Ohba H, Yasunaga M, Nonaka K, Takeuchi R, Nishi M, Sakuma N, Uchida H, Shinkai S, Fujiwara Y (2015). The effect of intergenerational programs on the mental health of elderly adults. Aging Ment Health.

[126] Marken DM, Howard JB (2014). Grandparents raising grandchildren: the influence of a late-life transition on occupational engagement. Physical Occupational Therapy Geriatrics.

[127] Rook KS, Sorkin DH (2016). Fostering social ties through a volunteer role: implications for older-adults' psychological health. Int J Aging Hum Dev.

[128] Chung JCC (2009). An intergenerational reminiscence programme for older adults with early dementia and youth volunteers: values and challenges. Scand J Caring Sci.

[129] Gaggioli A, Morganti L, Bonfiglio S, Scaratti C, Cipresso P, Serino S, Riva G (2014). Intergenerational group reminiscence: a potentially effective intervention to enhance elderly psychosocial wellbeing and to improve children's perception of aging. Educational Gerontol.

[130] Nicholson Jr NR, Shellman J (2013). Decreasing social isolation in older adults: effects of an empowerment intervention offered through the CARELINK program. Res Gerontol Nurs.

[131] Arthanat S, Vroman KG, Lysack C (2016). A home-based individualized information communication technology training program for older adults: a demonstration of effectiveness and value. Disabil Rehabil Assist Technol.

[132] Heyn Billipp S (2001). The psychosocial impact of interactive computer use within a vulnerable elderly population: a report on a randomized prospective trial in a home health care setting. Public Health Nurs.

[133] Blažun H, Saranto K, Kokol P, Vošner J (2012). Information and communication technology as a tool for improving physical and social activity of the elderly. NI 2012 (2012).

[134] Cotten SR, Anderson WA, McCullough BM (2013). Impact of internet use on loneliness and contact with others among older adults: cross-sectional analysis. J Med Internet Res.

[135] Delello JA, McWhorter RR (2017). Reducing the digital divide: connecting older adults to iPad technology. J Appl Gerontol.

[136] Fokkema T, Knipscheer K (2007). Escape loneliness by going digital: a quantitative and qualitative evaluation of a Dutch experiment in using ECT to overcome loneliness among older adults. Aging Ment Health.

[137] Jones RB, Ashurst EJ, Atkey J, Duffy B (2015). Older people going online: its value and before-after evaluation of volunteer support. J Med Internet Res.

[138] Lagana L, Garcia JJ (2013). The mental health impact of computer and internet training on a multi-ethnic sample of community-dwelling older adults: results of a pilot randomised controlled trial. Int J Biomed Sci.

[139] Mellor D, Firth L, Moore K (2008). Can the internet improve the well-being of the elderly?. Ageing Int.

[140] Shapira N, Barak A, Gal I (2007). Promoting older adults' well-being through internet training and use. Aging Ment Health.

[141] Slegers K, van Boxtel MP, Jolles J (2007). The effects of computer training and internet usage on the use of everyday technology by older adults: a randomized controlled study. Educ Gerontol.

[142] Slegers K, van Boxtel MP, Jolles J (2008). Effects of computer training and Internet usage on the well-being and quality of life of older adults: a randomized, controlled study. J Gerontol B Psychol Sci Soc Sci.

[143] White H, McConnell E, Clipp E, Branch LG, Sloane R, Pieper C, Box TL (2002). A randomized controlled trial of the psychosocial impact of providing internet training and access to older adults. Aging Ment Health.

[144] White H, McConnell E, Clipp E, Bynum L, Teague C, Navas L, Craven S, Halbrecht H (2016). Surfing the net in later life: a review of the literature and pilot study of computer use and quality of life. J Appl Gerontol.

[145] Winstead V, Anderson WA, Yost EA, Cotten SR, Warr A, Berkowsky RW (2013). You can teach an old dog new tricks: a qualitative analysis of how residents of senior living communities may use the web to overcome spatial and social barriers. J Appl Gerontol.

[146] Woodward AT, Freddolino PP, Blaschke-Thompson CM, Wishart DJ, Bakk L, Kobayashi R, Tupper C (2010). Technology and aging project: training outcomes and efficacy from a randomized field trial. Ageing Int.

[147] Ballantyne A, Trenwith L, Zubrinich S, Corlis M (2010). ‘I feel less lonely’: what older people say about participating in a social networking website. Qual Ageing Older Adult.

[148] Ballesteros S, Toril P, Mayas J, Reales J, Waterworth J (2014). An ICT-mediated social network in support of successful ageing. Gerontechnology.

[149] Larsson E, Nilsson I, Larsson Lund M (2013). Participation in social internet-based activities: five seniors' intervention processes. Scand J Occup Ther.

[150] Cattan M, Kime N, Bagnall A (2011). The use of telephone befriending in low level support for socially isolated older people--an evaluation. Health Soc Care Community.

[151] Evans RL, Werkhoven W, Fox HR (1982). Treatment of social isolation and loneliness in a sample of visually impaired elderly persons. Psychol Rep.

[152] Heller K, Thompson M, Trueba P, Hogg J, Vlachos-Weber I (1991). Peer support telephone dyads for elderly women: was this the wrong intervention?. Am J Community Psychol.

[153] Mahoney DF, Tarlow BJ, Jones RN (2003). Effects of an automated telephone support system on caregiver burden and anxiety: findings from the REACH for TLC intervention study. Gerontologist.

[154] Mountain GA, Hind D, Gossage-Worrall R, Walters SJ, Duncan R, Newbould L, Rex S, Jones C, Bowling A, Cattan M, Cairns A, Cooper C, Edwards RT, Goyder EC (2014). 'Putting Life in Years' (PLINY) telephone friendship groups research study: pilot randomised controlled trial. Trials.

[155] Savolainen L, Hanson E, Magnusson L, Gustavsson T (2008). An Internet-based videoconferencing system for supporting frail elderly people and their carers. J Telemed Telecare.

[156] Stewart M, Mann K, Jackson S, Downe-Wamboldt B, Bayers L, Slater M, Turner L (2010). Telephone support groups for seniors with disabilities. Can J Aging.

[157] Travers C, Bartlett H (2010). An exploratory study of Carers' and care staff's perspectives of silver memories—a unique radio program for older people. Activ Adapt Aging.

[158] Alaviani M, Khosravan S, Alami A, Moshki M (2015). The effect of a multi-strategy program on developing social behaviors based on Pender's health promotion model to prevent loneliness of old women referred to Gonabad urban health centers. Int J Community Based Nurs Midwifery.

[159] Andersson L (1985). Intervention against loneliness in a group of elderly women: an impact evaluation. Soc Sci Med.

[160] Bøen H, Dalgard OS, Johansen R, Nord E (2012). A randomized controlled trial of a senior centre group programme for increasing social support and preventing depression in elderly people living at home in Norway. BMC Geriatr.

[161] Constantino R (1988). Comparison of two group interventions for the bereaved. Image J Nurs Sch.

[162] Lökk J (1990). Emotional and social effects of a controlled intervention study in a day-care unit for elderly patients. Scand J Prim Health Care.

[163] Martina CM, Stevens NL (2006). Breaking the cycle of loneliness? Psychological effects of a friendship enrichment program for older women. Aging Ment Health.

[164] Ormsby J, Stanley M, Jaworski K (2010). Older men's participation in community-based men's sheds programmes. Health Soc Care Community.

[165] Stevens NL, Martina CM, Westerhof GJ (2006). Meeting the need to belong: predicting effects of a friendship enrichment program for older women. Gerontologist.

[166] Tilburg NS, van Tilburg T (2010). Stimulating friendship in later life: a strategy for reducing loneliness among older women. Educ Gerontol.

[167] Stewart M, Craig D, MacPherson K, Alexander S (2001). Promoting positive affect and diminishing loneliness of widowed seniors through a support intervention. Public Health Nurs.

[168] BARTLETT H, WARBURTON J, LUI C, PEACH L, CARROLL M (2012). Preventing social isolation in later life: findings and insights from a pilot Queensland intervention study. Ageing Soc.

[169] Crawford RD (1977). A Study of the Psychosocial and Sociological Effects That Being a Member of the Retired Senior Volunteer Program Has Upon Persons Aged Sixty and Over in Salt Lake County Utah.

[170] Iecovich E, Biderman A (2011). Attendance in adult day care centers and its relation to loneliness among frail older adults. Int Psychogeriatr.

[171] Mulry CM, Piersol CV (2014). The let's go program for community participation: a feasibility study. Physical Occupational Ther Geriatr.

[172] Collins CC, Benedict J (2006). Evaluation of a community-based health promotion program for the elderly: lessons from seniors can. Am J Health Promotion.

[173] Cox EO, Green KE, Hobart K, Jang LJ, Seo H (2007). Strengthening the late-life care process: effects of two forms of a care-receiver efficacy intervention. Gerontologist.

[174] Kremers IP, Steverink N, Albersnagel FA, Slaets JP (2006). Improved self-management ability and well-being in older women after a short group intervention. Aging Ment Health.

[175] Saito T, Kai I, Takizawa A (2012). Effects of a program to prevent social isolation on loneliness, depression, and subjective well-being of older adults: a randomized trial among older migrants in Japan. Arch Gerontol Geriatr.

[176] Butler SS (2006). Evaluating the senior companion program. J Gerontol Soc Work.

[177] Dickens AP, Richards SH, Hawton A, Taylor RS, Greaves CJ, Green C, Edwards R, Campbell JL (2011). An evaluation of the effectiveness of a community mentoring service for socially isolated older people: a controlled trial. BMC Public Health.

[178] Greaves CJ, Farbus L (2006). Effects of creative and social activity on the health and well-being of socially isolated older people: outcomes from a multi-method observational study. J R Soc Promot Health.

[179] Caserta MS, Lund DA (1993). Intrapersonal resources and the effectiveness of self-help groups for bereaved older adults. Gerontologist.

[180] Creswell JD, Irwin MR, Burklund LJ, Lieberman MD, Arevalo JM, Ma J, Breen EC, Cole SW (2012). Mindfulness-based stress reduction training reduces loneliness and pro-inflammatory gene expression in older adults: a small randomized controlled trial. Brain Behav Immun.

[181] Drentea P, Clay OJ, Roth DL, Mittelman MS (2006). Predictors of improvement in social support: five-year effects of a structured intervention for caregivers of spouses with Alzheimer's disease. Soc Sci Med.

[182] Liu SJ, Lin CJ, Chen YM, Huang XY (2007). The effects of reminiscence group therapy on self-esteem, depression, loneliness and life satisfaction of elderly people living alone. Mid Taiwan J Med.

[183] Rosen CE, Rosen S (1982). Evaluating an intervention program for the elderly. Community Ment Health J.

[184] Routasalo PE, Tilvis RS, Kautiainen H, Pitkala KH (2009). Effects of psychosocial group rehabilitation on social functioning, loneliness and well-being of lonely, older people: randomized controlled trial. J Adv Nurs.

[185] Clark F, Azen SP, Carlson M, Mandel D, LaBree L, Hay J, Zemke R, Jackson J, Lipson L (2001). Embedding health-promoting changes into the daily lives of independent-living older adults: long-term follow-up of occupational therapy intervention. J Gerontol B Psychol Sci Soc Sci.

[186] Clark F, Azen SP, Zemke R, Jackson J, Carlson M, Mandel D, Hay J (1997). Occupational therapy for independent-living older adults. JAMA.

[187] Clark F, Jackson J, Carlson M, Chou C, Cherry BJ, Jordan-Marsh M, Knight BG, Mandel D, Blanchard J, Granger DA, Wilcox RR, Lai MY, White B, Hay J, Lam C, Marterella A, Azen SP (2012). Effectiveness of a lifestyle intervention in promoting the well-being of independently living older people: results of the Well Elderly 2 Randomised Controlled Trial. J Epidemiol Community Health.

[188] de Craen AJM, Gussekloo J, Blauw GJ, Willems CG, Westendorp RGJ (2006). Randomised controlled trial of unsolicited occupational therapy in community-dwelling elderly people: the LOTIS trial. PLoS Clin Trials.

[189] Girdler SJ, Boldy DP, Dhaliwal SS, Crowley M, Packer TL (2010). Vision self-management for older adults: a randomised controlled trial. Br J Ophthalmol.

[190] Graff MJ, Adang EM, Vernooij-Dassen MJ, Dekker J, Jönsson L, Thijssen M, Hoefnagels WH, Rikkert MG (2008). Community occupational therapy for older patients with dementia and their care givers: cost effectiveness study. BMJ.

[191] Graff MJL, Vernooij-Dassen MJ, Thijssen M, Dekker J, Hoefnagels WH, Olderikkert MG (2007). Effects of community occupational therapy on quality of life, mood, and health status in dementia patients and their caregivers: a randomized controlled trial. J Gerontol A Biol Sci Med Sci.

[192] Graff MJ, Vernooij-Dassen MJ, Thijssen M, Dekker J, Hoefnagels WH, Rikkert MG (2006). Community based occupational therapy for patients with dementia and their care givers: randomised controlled trial. BMJ.

[193] Hay J, LaBree L, Luo R, Clark F, Carlson M, Mandel D, Zemke R, Jackson J, Azen SP (2002). Cost-effectiveness of preventive occupational therapy for independent-living older adults. J Am Geriatr Soc.

[194] Jackson J, Carlson M, Mandel D, Zemke R, Clark F (1998). Occupation in lifestyle redesign: the well elderly study occupational therapy program. Am J Occup Ther.

[195] Jackson J, Mandel D, Blanchard J, Carlson M, Cherry B, Azen S, Chou C, Jordan-Marsh M, Forman T, White B, Granger D, Knight B, Clark F (2009). Confronting challenges in intervention research with ethnically diverse older adults: the USC Well Elderly II Trial. Clin Trials.

[196] Leland NE, Fogelberg D, Sleight A, Mallinson T, Vigen C, Blanchard J, Carlson M, Clark F (2016). Napping and nighttime sleep: findings from an occupation-based intervention. Am J Occup Ther.

[197] Low LF, Baker JR, Harrison F, Jeon YH, Haertsch M, Camp C, Skropeta M (2015). The lifestyle engagement activity program (LEAP): implementing social and recreational activity into case-managed home care. J Am Med Dir Assoc.

[198] Matuska K, Giles-Heinz A, Flinn N, Neighbor M, Bass-Haugen J (2003). Outcomes of a pilot occupational therapy wellness program for older adults. Am J Occup Ther.

[199] Melis RJ, van Eijken MI, Teerenstra S, van Achterberg T, Parker SG, Borm GF, van de Lisdonk EH, Wensing M, Rikkert MG (2008). A randomized study of a multidisciplinary program to intervene on geriatric syndromes in vulnerable older people who live at home (Dutch EASYcare Study). J Gerontol A Biol Sci Med Sci.

[200] Mountain G, Mozley C, Craig C, Ball L (2008). Occupational therapy led health promotion for older people: feasibility of the lifestyle matters programme. Br J Occup Ther.

[201] Johansson A, Björklund A (2016). The impact of occupational therapy and lifestyle interventions on older persons' health, well-being, and occupational adaptation. Scand J Occup Ther.

[202] Alnahdi G (2014). Assistive technology in special education and the universal design for learning. Turkish Online J Educ Technol.

[203] Bogat GA, Jason LA (1995). An evaluation of two visiting programs for elderly community residents. Int J Aging Hum Dev.

[204] Clarke M, Clarke SJ, Jagger C (1992). Social intervention and the elderly: a randomized controlled trial. Am J Epidemiol.

[205] Hall N, de Beck P, Johnson D, Mackinnon K, Gutman G, Glick N (2010). Randomized trial of a health promotion program for frail elders. Can J Aging.

[206] Mulligan MA, Bennett R (1977). Assessment of mental health and social problems during multiple friendly visits: the development and evaluation of a friendly visiting program for the isolated elderly. Int J Aging Hum Dev.

[207] van Rossum E, Frederiks CM, Philipsen H, Portengen K, Wiskerke J, Knipschild P (1993). Effects of preventive home visits to elderly people. BMJ.

[208] Winter L, Gitlin LN (2006). Evaluation of a telephone-based support group intervention for female caregivers of community-dwelling individuals with dementia. Am J Alzheimers Dis Other Demen.

[209] Coleman EA, Tulman L, Samarel N, Wilmoth MC, Rickel L, Rickel M, Stewart CB (2005). The effect of telephone social support and education on adaptation to breast cancer during the year following diagnosis. Oncol Nurs Forum.

[210] Evans RL, Jaureguy BM (1982). Phone therapy outreach for blind elderly. Gerontologist.

[211] Hartke RJ, King RB (2015). Telephone group intervention for older stroke caregivers. Topics Stroke Rehabil.

[212] Morrow-Howell N, Becker-Kemppainen S, Judy L (2016). Evaluating an intervention for the elderly at increased risk of suicide. Res Soc Work Pract.

[213] Bond GE, Burr RL, Wolf FM, Feldt K (2010). The effects of a web-based intervention on psychosocial well-being among adults aged 60 and older with diabetes: a randomized trial. Diabetes Educ.

[214] Brennan PF, Moore SM, Smyth KA (1995). The effects of a special computer network on caregivers of persons with alzheimer's disease. Nurs Res.

[215] Westlake C, Evangelista LS, Strömberg A, Ter-Galstanyan A, Vazirani S, Dracup K (2007). Evaluation of a web-based education and counseling pilot program for older heart failure patients. Prog Cardiovasc Nurs.

[216] McEwan RT, Davison N, Forster DP, Pearson P, Stirling E (1990). Screening elderly people in primary care: a randomized controlled trial. Br J Gen Pract.

[217] Sørensen KH, Sivertsen J (1988). Follow-up three years after intervention to relieve unmet medical and social needs of old people. Compr Gerontol B.

[218] Sørensen KH, Sivertsen J, Schroll M, Gjørup S (1982). A socio-medical population study of the elderly in the city of Copenhagen 1978/79. General presentation and methodological aspects. Dan Med Bull.

[219] Cole KM, Gawlinski A, Steers N, Kotlerman J (2007). Animal-assisted therapy in patients hospitalized with heart failure. Am J Crit Care.

[220] Holcomb R, Jendro C, Weber B, Nahan U (2015). Use of an aviary to relieve depression in elderly males. Anthrozoös.

[221] Jessen J, Cardiello F, Baun MM (1996). Avian companionship in alleviation of depression, loneliness, and low morale of older adults in skilled rehabilitation units. Psychol Rep.

[222] Ko HJ, Youn CH, Kim SH, Kim SY (2016). Effect of pet insects on the psychological health of community-dwelling elderly people: a single-blinded, randomized, controlled trial. Gerontology.

[223] Krause-Parello CA, Kolassa J (2016). Pet therapy: enhancing social and cardiovascular wellness in community dwelling older adults. J Community Health Nurs.

[224] Riddick CC (2008). Health, aquariums, and the non-institutionalized elderly. Marriage Family Rev.

[225] Broadbent E, Peri K, Kerse N, Jayawardena C, Kuo I, Datta C (2014). Robots in older people’s homes to improve medication adherence and quality of life: a randomised cross-over trial. Social Robotics.

[226] Kanamori M, Suzuki M, Oshiro H, Tanaka M, Inoguchi T, Takasugi H, Saito Y, Yokoyama T (2003). Pilot study on improvement of quality of life among elderly using a pet-type robot. Proceedings 2003 IEEE International Symposium on Computational Intelligence in Robotics and Automation. Computational Intelligence in Robotics and Automation for the New Millennium (Cat. No.03EX694).

[227] Machesney D, Wexler SS, Chen T, Coppola JF (2014). Gerontechnology Companion: virutal pets for dementia patients. Proceedings of the IEEE Long Island Systems, Applications and Technology (LISAT) Conference 2014.

[228] Sebastian J, Hsu Y, Lu J (2014). Creation of a 'Caricature Robot' for social inclusion of older adults. Gerontechnology.

[229] Tamura T, Yonemitsu S, Itoh A, Oikawa D, Kawakami A, Higashi Y, Fujimooto T, Nakajima K (2004). Is an entertainment robot useful in the care of elderly people with severe dementia?. J Gerontol A Biol Sci Med Sci.

[230] Tanaka M, Ishii A, Yamano E, Ogikubo H, Okazaki M, Kamimura K, Konishi Y, Emoto S, Watanabe Y (2012). Effect of a human-type communication robot on cognitive function in elderly women living alone. Med Sci Monit.

[231] Clift S, Skingley A, Coulton S, Rodriguez J (2012). A controlled evaluation of the health benefits of a participative community singing programme for older people. Arts & Health South West.

[232] Coffman D (2009). Survey of new horizons international music association musicians. Int J Community Music.

[233] Cohen GD, Perlstein S, Chapline J, Kelly J, Firth KM, Simmens S (2007). The impact of professionally conducted cultural programs on the physical health, mental health, and social functioning of older adults—2-year results. J Aging Humanities Arts.

[234] Davidson JW, McNamara B, Rosenwax L, Lange A, Jenkins S, Lewin G (2014). Evaluating the potential of group singing to enhance the well-being of older people. Australas J Ageing.

[235] Hillman S (2002). Participatory singing for older people: a perception of benefit. Health Educ.

[236] Kattenstroth JC, Kalisch T, Holt S, Tegenthoff M, Dinse HR (2013). Six months of dance intervention enhances postural, sensorimotor, and cognitive performance in elderly without affecting cardio-respiratory functions. Front Aging Neurosci.

[237] Koga M (2001). The music making and wellness project. Am Music Teacher.

[238] Solé C, Mercadal-Brotons M, Gallego S, Riera M (2010). Contributions of music to aging adults' quality of life. J Music Ther.

[239] Teater B, Baldwin M (2012). Singing for successful ageing: the perceived benefits of participating in the golden oldies community-arts programme. Brit J Social Work.

[240] Verghese J, Lipton RB, Katz MJ, Hall CB, Derby CA, Kuslansky G, Ambrose AF, Sliwinski M, Buschke H (2003). Leisure activities and the risk of dementia in the elderly. N Engl J Med.

[241] Yap AF, Kwan YH, Tan CS, Ibrahim S, Ang SB (2017). Rhythm-centred music making in community living elderly: a randomized pilot study. BMC Complement Altern Med.

[242] Cohen GD, Perlstein S, Chapline J, Kelly J, Firth KM, Simmens S (2006). The impact of professionally conducted cultural programs on the physical health, mental health, and social functioning of older adults. Gerontologist.

[243] Arnetz BB, Theorell T (1983). Psychological, sociological and health behaviour aspects of a long term activation programme for institutionalized elderly people. Soc Sci Med.

[244] Baumgarten M, Thomas D, de Courval LP, Infante-Rivard C (1988). Evaluation of a mutual help network for the elderly residents of planned housing. Psychol Aging.

[245] Gleibs IH, Haslam C, Jones JM, Alexander Haslam S, McNeill J, Connolly H (2011). No country for old men? The role of a 'Gentlemen's Club' in promoting social engagement and psychological well-being in residential care. Aging Ment Health.

[246] Hemingway A, Jack E (2013). Reducing social isolation and promoting well being in older people. Quality Ageing Older Adult.

[247] Pettigrew S, Roberts M (2008). Addressing loneliness in later life. Aging Ment Health.

[248] Tse T, Linsey H (2005). Adult day groups: addressing older people's needs for activity and companionship. Australas J Ageing.

[249] Valadez AA, Lumadue C, Gutierrez B, de Vries-Kell S (2006). Las Comadres and adult day care centers: the perceived impact of socialization on mental wellness. J Aging Stud.

[250] Moody E, Phinney A (2012). A community-engaged art program for older people: fostering social inclusion. Can J Aging.

[251] Agmon M, Perry C, Phelan E, Demiris G, Nguyen H (2011). A pilot study of Wii Fit exergames to improve balance in older adults. J Geriatr Phys Ther.

[252] Chao YY, Musanti R, Zha P, Katigbak C (2018). The feasibility of an exergaming program in underserved older African Americans. West J Nurs Res.

[253] Jung Y, Li KJ, Janissa NS, Gladys WL, Lee KM (2009). Games for a better life: effects of playing Wii games on the well-being of seniors in a long-term care facility. Proceedings of the Sixth Australasian Conference on Interactive Entertainment.

[254] Rendon AA, Lohman EB, Thorpe D, Johnson EG, Medina E, Bradley B (2012). The effect of virtual reality gaming on dynamic balance in older adults. Age Ageing.

[255] Rosenberg D, Depp CA, Vahia IV, Reichstadt J, Palmer BW, Kerr J, Norman G, Jeste DV (2010). Exergames for subsyndromal depression in older adults: a pilot study of a novel intervention. Am J Geriatric Psychiatry.

[256] Wollersheim D, Merkes M, Shields N, Liamputtong P, Wallis L, Reynolds F (2010). Physical and psychosocial effects of Wii video game use among older women. Aus J Emerging Technologies Soc.

[257] Xu X, Li J, Pham TP, Salmon CT, Theng Y (2016). Improving psychosocial well-being of older adults through exergaming: the moderation effects of intergenerational communication and age cohorts. Games Health J.

[258] Hopman-Rock M, Westhoff MH (2002). Development and evaluation of “aging well and healthily”: a health-education and exercise program for community-living older adults. J Aging Physical Activity.

[259] Kamegaya T, Araki Y, Kigure H, Yamaguchi H, Long-Term-Care Prevention Team of Maebashi City (2014). Twelve-week physical and leisure activity programme improved cognitive function in community-dwelling elderly subjects: a randomized controlled trial. Psychogeriatrics.

[260] McAuley E, Blissmer B, Marquez DX, Jerome GJ, Kramer AF, Katula J (2000). Social relations, physical activity, and well-being in older adults. Prev Med.

[261] Seino S, Nishi M, Murayama H, Narita M, Yokoyama Y, Nofuji Y, Taniguchi Y, Amano H, Kitamura A, Shinkai S (2017). Effects of a multifactorial intervention comprising resistance exercise, nutritional and psychosocial programs on frailty and functional health in community-dwelling older adults: a randomized, controlled, cross-over trial. Geriatr Gerontol Int.

[262] Beer J, Takayama L (2011). Mobile remote presence systems for older adults: acceptance, benefits, and concerns. Proceedings of the 6th international conference on Human-robot interaction.

[263] Liang A, Piroth I, Robinson H, MacDonald B, Fisher M, Nater UM, Skoluda N, Broadbent E (2017). A pilot randomized trial of a companion robot for people with dementia living in the community. J Am Med Dir Assoc.

[264] Seelye AM, Wild KV, Larimer N, Maxwell S, Kearns P, Kaye JA (2012). Reactions to a remote-controlled video-communication robot in seniors' homes: a pilot study of feasibility and acceptance. Telemed J E Health.

[265] Torta E, Werner F, Johnson DO, Juola JF, Cuijpers RH, Bazzani M, Oberzaucher J, Lemberger J, Lewy H, Bregman J (2014). Evaluation of a small socially-assistive humanoid robot in intelligent homes for the care of the elderly. J Intell Robot Syst.

[266] Saito T, Shibata T, Wada K, Tanie K (2004). Change of stress reaction of the elderly by interaction with robot seal in health services facility for the aged. Proceedings of the Joint 2nd International Conference on Soft Computing and Intelligent Systems and 5th International Symposium on Advanced Intelligent Systems (SCIS&ISIS).

[267] Saito T, Shibata T, Wada K, Tanie K (2003). Relationship between interaction with the mental commit robot and change of stress reaction of the elderly. Proceedings 2003 IEEE International Symposium on Computational Intelligence in Robotics and Automation. Computational Intelligence in Robotics and Automation for the New Millennium (Cat. No.03EX694).

[268] Shibata T, Wada K (2008). Robot therapy at elder care institutions: effects of long-term interaction with seal robots. The Engineering Handbook of Smart Technology for Aging, Disability, and Independence.

[269] Wada K, Shibata T, Saito T, Tanie K (2002). Effects of robot assisted activity for elderly people at day service center and analysis of its factors. Proceedings of the 4th World Congress on Intelligent Control and Automation (Cat. No.02EX527).

[270] Wada K, Shibata T, Saito T, Tanie K (2003). Relationship between familiarity with mental commit robot and psychological effects to elderly people by robot assisted activity. Proceedings 2003 IEEE International Symposium on Computational Intelligence in Robotics and Automation. Computational Intelligence in Robotics and Automation for the New Millennium (Cat. No.03EX694).

[271] Wada K, Shibata T, Saito T, Tanie K (2003). Effects of robot assisted activity to elderly people who stay at a health service facility for the aged. Proceedings 2003 IEEE/RSJ International Conference on Intelligent Robots and Systems (IROS 2003) (Cat. No.03CH37453).

[272] Bartsch DA, Rodgers VK (2009). Senior reach outcomes in comparison with the Spokane Gatekeeper program. Care Manag J.

[273] Bartsch DA, Rodgers VK, Strong D (2013). Outcomes of senior reach gatekeeper referrals: comparison of the Spokane gatekeeper program, Colorado Senior Reach, and Mid-Kansas Senior Outreach. Care Manag J.

[274] Leung GT, Leung KF, Lam LC (2011). Classification of late-life leisure activities among elderly Chinese in Hong Kong. East Asian Arch Psychiatry.

[275] Levasseur M, Richard L, Gauvin L, Raymond É (2010). Inventory and analysis of definitions of social participation found in the aging literature: proposed taxonomy of social activities. Soc Sci Med.

[276] Caroli MG, Fracassi E, Maiolini R, Carnini Pulino S (2018). Exploring social innovation components and attributes: a taxonomy proposal. J Social Entrepreneurship.

[277] MacCourt P Social isolation of seniors - Volume 1: understanding the issue and finding solutions. Government of Canada.

[278] Chatterjee HJ, Camic PM, Lockyer B, Thomson LJ (2017). Non-clinical community interventions: a systematised review of social prescribing schemes. Arts Health.

[279] Bickerdike L, Booth A, Wilson PM, Farley K, Wright K (2017). Social prescribing: less rhetoric and more reality. A systematic review of the evidence. BMJ Open.

[280] Sen K, Prybutok G, Prybutok V (2022). The use of digital technology for social wellbeing reduces social isolation in older adults: a systematic review. SSM Popul Health.

[281] Adams KB, Sanders S, Auth EA (2004). Loneliness and depression in independent living retirement communities: risk and resilience factors. Aging Ment Health.

[282] Ayalon L, Shiovitz-Ezra S, Palgi Y (2013). Associations of loneliness in older married men and women. Aging Ment Health.

[283] Béland F, Zunzunegui MV, Alvarado B, Otero A, del Ser T (2005). Trajectories of cognitive decline and social relations. J Gerontol B Psychol Sci Soc Sci.

[284] Chalise HN, Kai I, Saito T (2010). Social support and its correlation with loneliness: a cross-cultural study of Nepalese older adults. Int J Aging Hum Dev.

[285] Chi I, Yuan L, Meng T (2013). Multidimensional needs assessment for low-income Chinese seniors in subsidized housing in Los Angeles. Seniors Housing Care J.

[286] Cohen-Mansfield J, Shmotkin D, Goldberg S (2009). Loneliness in old age: longitudinal changes and their determinants in an Israeli sample. Int Psychogeriatr.

[287] Dong X, Chang E, Wong E, Simon M (2012). Perception and negative effect of loneliness in a Chicago Chinese population of older adults. Arch Gerontol Geriatr.

[288] Dong X, Li Y, Simon MA (2014). Social engagement among U.S. Chinese older adults--findings from the PINE Study. J Gerontol A Biol Sci Med Sci.

[289] Fry PS, Debats DL (2016). Self-efficacy beliefs as predictors of loneliness and psychological distress in older adults. Int J Aging Hum Dev.

[290] Gee EM (2000). Living arrangements and quality of life among Chinese Canadian elders. Social Indicator Res.

[291] Giles LC, Anstey KJ, Walker RB, Luszcz MA (2012). Social networks and memory over 15 years of followup in a cohort of older Australians: results from the Australian longitudinal study of ageing. J Aging Res.

[292] Havens B, Hall M, Sylvestre G, Jivan T (2004). Social isolation and loneliness: differences between older rural and urban Manitobans. Can J Aging.

[293] Hill TD, Burdette AM, Angel JL, Angel RJ (2006). Religious attendance and cognitive functioning among older Mexican Americans. J Gerontol B Psychol Sci Soc Sci.

[294] Iliffe S, Kharicha K, Harari D, Swift C, Gillmann G, Stuck AE (2007). Health risk appraisal in older people 2: the implications for clinicians and commissioners of social isolation risk in older people. Br J Gen Pract.

[295] IP D, LUI CW, CHUI WH (2007). Veiled entrapment: a study of social isolation of older Chinese migrants in Brisbane, Queensland. Ageing Soc.

[296] Kim BJ, Sangalang CC, Kihl T (2012). Effects of acculturation and social network support on depression among elderly Korean immigrants. Aging Ment Health.

[297] Kuyper L, Fokkema T (2010). Loneliness among older lesbian, gay, and bisexual adults: the role of minority stress. Arch Sex Behav.

[298] Losada A, Márquez-González M, García-Ortiz L, Gómez-Marcos MA, Fernández-Fernández V, Rodríguez-Sánchez E (2012). Loneliness and mental health in a representative sample of community-dwelling Spanish older adults. J Psychol.

[299] Martin-Matthews A, Tong CE, Rosenthal CJ, McDonald L (2013). Ethno-cultural diversity in the experience of widowhood in later life: Chinese widows in Canada. J Aging Stud.

[300] Mui AC (1996). Depression among elderly Chinese immigrants: an exploratory study. Social Work.

[301] Mui AC (2008). Living alone and depression among older Chinese immigrants. J Gerontological Social Work.

[302] Newall NE, Chipperfield JG, Clifton RA, Perry RP, Swift AU, Ruthig JC (2009). Causal beliefs, social participation, and loneliness among older adults: a longitudinal study. J Social Personal Relationship.

[303] Ng CF, Northcott HC (2013). Living arrangements and loneliness of South Asian immigrant seniors in Edmonton, Canada. Ageing Soc.

[304] Routasalo PE, Savikko N, Tilvis RS, Strandberg TE, Pitkälä KH (2006). Social contacts and their relationship to loneliness among aged people - a population-based study. Gerontology.

[305] Savikko N, Routasalo P, Tilvis RS, Strandberg TE, Pitkälä KH (2005). Predictors and subjective causes of loneliness in an aged population. Arch Gerontol Geriatr.

[306] Shiovitz-Ezra S, Leitsch SA (2010). The role of social relationships in predicting loneliness: the national social life, health, and aging project. Social Work Res.

[307] Tam S, Neysmith S (2006). Disrespect and isolation: elder abuse in Chinese communities. Can J Aging.

[308] Theeke LA (2009). Predictors of loneliness in U.S. adults over age sixty-five. Arch Psychiatr Nurs.

[309] Theeke LA (2010). Sociodemographic and health-related risks for loneliness and outcome differences by loneliness status in a sample of U.S. older adults. Res Gerontol Nurs.

[310] Victor CR, Scambler SJ, Bowling A, Bond J (2005). The prevalence of, and risk factors for, loneliness in later life: a survey of older people in Great Britain. Ageing Soc.

[311] Wong ST, Yoo GJ, Stewart AL (2007). An empirical evaluation of social support and psychological well-being in older Chinese and Korean immigrants. Ethn Health.

[312] Alma MA, van der Mei SF, Feitsma WN, Groothoff JW, van Tilburg TG, Suurmeijer TP (2011). Loneliness and self-management abilities in the visually impaired elderly. J Aging Health.

[313] Burns RA, Browning CJ, Kendig HL (2015). Examining the 16-year trajectories of mental health and wellbeing through the transition into widowhood. Int Psychogeriatr.

[314] Cacioppo JT, Hawkley LC, Thisted RA (2010). Perceived social isolation makes me sad: 5-year cross-lagged analyses of loneliness and depressive symptomatology in the Chicago Health, Aging, and Social Relations Study. Psychol Aging.

[315] Carr D, Sonnega J, Nesse RM, House JS (2014). Do special occasions trigger psychological distress among older bereaved spouses? An empirical assessment of clinical wisdom. J Gerontol B Psychol Sci Soc Sci.

[316] Cheng P, Jin Y, Sun H, Tang Z, Zhang C, Chen Y, Zhang Q, Zhang Q, Huang F (2015). Disparities in prevalence and risk indicators of loneliness between rural empty nest and non-empty nest older adults in Chizhou, China. Geriatr Gerontol Int.

[317] Cohen-Mansfield J, Parpura-Gill A (2006). Loneliness in older persons: a theoretical model and empirical findings. Int Psychogeriatrics.

[318] Drennan J, Treacy M, Butler M, Byrne A, Fealy G, Frazer K, Irving K (2008). The experience of social and emotional loneliness among older people in Ireland. Ageing Soc.

[319] Fokkema T, de Jong Gierveld J, Dykstra PA (2012). Cross-national differences in older adult loneliness. J Psychol.

[320] Ghesquiere A, Shear MK, Duan N (2013). Outcomes of bereavement care among widowed older adults with complicated grief and depression. J Prim Care Community Health.

[321] Greenfield EA, Russell D (2010). Identifying living arrangements that heighten risk for loneliness in later life. J Appl Gerontol.

[322] Hawkley LC, Hughes ME, Waite LJ, Masi CM, Thisted RA, Cacioppo JT (2008). From social structural factors to perceptions of relationship quality and loneliness: the Chicago health, aging, and social relations study. J Gerontol B Psychol Sci Soc Sci.

[323] Jeon GS, Jang SN, Kim DS, Cho SI (2013). Widowhood and depressive symptoms among Korean elders: the role of social ties. J Gerontol B Psychol Sci Soc Sci.

[324] Palgi Y, Shrira A, Ben-Ezra M, Shiovitz-Ezra S, Ayalon L (2012). Self- and other-oriented potential lifetime traumatic events as predictors of loneliness in the second half of life. Aging Ment Health.

[325] Panagiotopoulos G, Walker R, Luszcz M (2013). A comparison of widowhood and well-being among older Greek and British-Australian migrant women. J Aging Stud.

[326] Paúl C, Ribeiro O (2009). Predicting loneliness in old people living in the community. Rev Clin Gerontol.

[327] Pinquart M, Sörensen S (2001). Gender differences in self-concept and psychological well-being in old age: a meta-analysis. J Gerontol B Psychol Sci Soc Sci.

[328] Aartsen M, Jylhä M (2011). Onset of loneliness in older adults: results of a 28 year prospective study. Eur J Ageing.

[329] Agrawal G, Keshri K (2014). Morbidity patterns and health care seeking behavior among older widows in India. PLoS One.

[330] Alpass FM, Neville S (2003). Loneliness, health and depression in older males. Aging Ment Health.

[331] Brummett BH, Barefoot JC, Siegler IC, Clapp-Channing NE, Lytle BL, Bosworth HB, Williams RB, Mark DB (2001). Characteristics of socially isolated patients with coronary artery disease who are at elevated risk for mortality. Psychosom Med.

[332] DiGiacomo M, Lewis J, Nolan MT, Phillips J, Davidson PM (2013). Health transitions in recently widowed older women: a mixed methods study. BMC Health Serv Res.

[333] Gould CE, Shah S, Brunskill SR, Brown K, Oliva NL, Hosseini C, Bauer E, Huh JW (2017). RESOLV: development of a telephone-based program designed to increase socialization in older veterans. Educational Gerontol.

[334] Han J, Richardson VE (2010). The relationship between depression and loneliness among homebound older persons: does spirituality moderate this relationship?. J Religion Spirituality Social Work Social Thought.

[335] Hyde C, Ward B, Horsfall J, Winder G (1999). Older women's experience of living with chronic leg ulceration. Int J Nurs Pract.

[336] Kramer SE, Kapteyn TS, Kuik DJ, Deeg DJ (2002). The association of hearing impairment and chronic diseases with psychosocial health status in older age. J Aging Health.

[337] Kuwert P, Knaevelsrud C, Pietrzak RH (2014). Loneliness among older veterans in the United States: results from the National Health and Resilience in Veterans Study. Am J Geriatr Psychiatry.

[338] La Grow S, Alpass F, Stephens C (2009). Economic standing, health status and social isolation among visually impaired persons aged 55 to 70 in New Zealand. J Optometry.

[339] La Grow S, Neville S, Alpass F, Rodgers V (2012). Loneliness and self-reported health among older persons in New Zealand. Australas J Ageing.

[340] Liu LJ, Guo Q (2007). Loneliness and health-related quality of life for the empty nest elderly in the rural area of a mountainous county in China. Qual Life Res.

[341] Marshall GL (2011). Predictors Associated With Late-life Depressive Symptoms Among Older Black Americans.

[342] Li J, Dong Q, Liu J-J, Dong Y-H, Yang L-S, Ye D, Huang F (2010). [Sleep and quality of life among rural elderly in Anhui province]. Zhonghua Liu Xing Bing Xue Za Zhi.

[343] Paul C, Ayis S, Ebrahim S (2006). Psychological distress, loneliness and disability in old age. Psychol Health Med.

[344] Perkins JM, Lee HY, James KS, Oh J, Krishna A, Heo J, Lee JK, Subramanian SV (2016). Marital status, widowhood duration, gender and health outcomes: a cross-sectional study among older adults in India. BMC Public Health.

[345] Prieto-Flores ME, Forjaz MJ, Fernandez-Mayoralas G, Rojo-Perez F, Martinez-Martin P (2011). Factors associated with loneliness of noninstitutionalized and institutionalized older adults. J Aging Health.

[346] Ramage-Morin PL (2016). Hearing difficulties and feelings of social isolation among Canadians aged 45 or older. Health Rep.

[347] Schnittger RI, Wherton J, Prendergast D, Lawlor BA (2012). Risk factors and mediating pathways of loneliness and social support in community-dwelling older adults. Aging Ment Health.

[348] Sjöberg L, Östling S, Falk H, Sundh V, Waern M, Skoog I (2013). Secular changes in the relation between social factors and depression: a study of two birth cohorts of Swedish septuagenarians followed for 5 years. J Affect Disord.

[349] Sung YK, Li L, Blake C, Betz J, Lin FR (2016). Association of hearing loss and loneliness in older adults. J Aging Health.

[350] Tiedt AD (2010). The gender gap in depressive symptoms among Japanese elders: evaluating social support and health as mediating factors. J Cross Cult Gerontol.

[351] Tomioka K, Ikeda H, Hanaie K, Morikawa M, Iwamoto J, Okamoto N, Saeki K, Kurumatani N (2013). The Hearing Handicap Inventory for Elderly-Screening (HHIE-S) versus a single question: reliability, validity, and relations with quality of life measures in the elderly community, Japan. Qual Life Res.

[352] Weinstein BE, Ventry IM (1982). Hearing impairment and social isolation in the elderly. J Speech Hear Res.

[353] Wells TS, Nickels LD, Rush SR, Musich SA, Wu L, Bhattarai GR, Yeh CS (2020). Characteristics and health outcomes associated with hearing loss and hearing aid use among older adults. J Aging Health.

[354] Wu ZQ, Sun L, Sun YH, Zhang XJ, Tao FB, Cui GH (2010). Correlation between loneliness and social relationship among empty nest elderly in Anhui rural area, China. Aging Ment Health.

[355] Zhou J, Hearst N (2016). Health-related quality of life of among elders in rural China: the effect of widowhood. Qual Life Res.

[356] La Grow SJ, Towers A, Yeung P, Alpass F, Stephens C (2015). The relationship between loneliness and perceived quality of life among older persons with visual impairments. J Visual Impairment Blindness.

[357] Luo Y, Hawkley LC, Waite LJ, Cacioppo JT (2012). Loneliness, health, and mortality in old age: a national longitudinal study. Soc Sci Med.

[358] Baumbusch JL (2004). Unclaimed treasures: older women's reflections on lifelong singlehood. J Women Aging.

[359] Choudhry U (2001). Uprooting and resettlement experiences of South Asian immigrant women. West J Nurs Res.

[360] de Jong Gierveld J, van der Pas S, Keating N (2015). Loneliness of older immigrant groups in Canada: effects of ethnic-cultural background. J Cross Cult Gerontol.

[361] Harrison T, Kahn DL, Hsu M (2016). A hermeneutic phenomenological study of widowhood for African-American women. Omega (Westport).

[362] Heikkinen SJ (2011). Exclusion of older immigrants from the former Soviet Union to Finland: the meaning of intergenerational relationships. J Cross Cult Gerontol.

[363] Heylen L (2010). The older, the lonelier? Risk factors for social loneliness in old age. Ageing So.

[364] Jacob SR (1996). The grief experience of older women whose husbands had hospice care. J Adv Nurs.

[365] Kim O (1999). Predictors of loneliness in elderly Korean immigrant women living in the United States of America. J Adv Nurs.

[366] Lauder W, Mummery K, Jones M, Caperchione C (2006). A comparison of health behaviours in lonely and non-lonely populations. Psychol Health Med.

[367] Li WW (2012). Art in health and identity: visual narratives of older Chinese immigrants to New Zealand. Arts Health.

[368] Luggen AS, Rini AG (1995). Assessment of social networks and isolation in community-based elderly men and women. Geriatr Nurs.

[369] Park HJ, Kim CG (2013). Ageing in an inconvenient paradise: the immigrant experiences of older Korean people in New Zealand. Australas J Ageing.

[370] Smith TF, Hirdes JP (2008). Predicting social isolation among geriatric psychiatry patients. Int Psychogeriatrics.

[371] Spahni S, Morselli D, Perrig-Chiello P, Bennett KM (2015). Patterns of psychological adaptation to spousal bereavement in old age. Gerontology.

[372] Victor CR, Bowling A (2012). A longitudinal analysis of loneliness among older people in Great Britain. J Psychol.

[373] Wenger GC, Burholt V (2004). Changes in levels of social isolation and loneliness among older people in a rural area: a twenty-year longitudinal study. Can J Aging.

[374] Zettel LA, Rook KS (2004). Substitution and compensation in the social networks of older widowed women. Psychol Aging.

[375] Xu L, Li Y, Min J, Chi I (2017). Worry about not having a caregiver and depressive symptoms among widowed older adults in China: the role of family support. Aging Ment Health.

[376] Krist AH, Tong ST, Aycock RA, Longo DR (2017). Engaging patients in decision-making and behavior change to promote prevention. Inform Services Use.

[377] Cosco TD, Fortuna K, Wister A, Riadi I, Wagner K, Sixsmith A (2021). COVID-19, social isolation, and mental health among older adults: a digital catch-22. J Med Internet Res.

[378] Popay J, Kowarzik U, Mallinson S, Mackian S, Barker J (2007). Social problems, primary care and pathways to help and support: addressing health inequalities at the individual level. Part I: the GP perspective. J Epidemiol Community Health.

[379] Hendler J (2004). Frequently Asked Questions on W3C's Web Ontology Language (OWL). W3C.

[380] Noy NF, McGuinness DL (2001). Ontology development 101: a guide to creating your first ontology. BibSonomy.

[381] Bechhofer S, van Harmelen F, Hendler J, Horrocks I, McGuinness DL, Patel-Schneider PF, Stein LA (2004). OWL web ontology language reference. W3C Recommendation.

[382] Smith B, Ashburner M, Rosse C, Bard J, Bug W, Ceusters W, Goldberg LJ, Eilbeck K, Ireland A, Mungall CJ, Consortium O, Leontis N, Rocca-Serra P, Ruttenberg A, Sansone S-A, Scheuermann RH, Shah N, Whetzel PL, Lewis S (2007). The OBO Foundry: coordinated evolution of ontologies to support biomedical data integration. Nat Biotechnol.

[383] Principle: commitment To collaboration (principle 10). OBO Foundry.

[384] Wilkinson MD, Dumontier M, Aalbersberg IJ, Appleton G, Axton M, Baak A, Blomberg N, Boiten J, da Silva Santos LB, Bourne PE, Bouwman J, Brookes AJ, Clark T, Crosas M, Dillo I, Dumon O, Edmunds S, Evelo CT, Finkers R, Gonzalez-Beltran A, Gray AJ, Groth P, Goble C, Grethe JS, Heringa J, 't Hoen P, Hooft R, Kuhn T, Kok R, Kok J, Lusher SJ, Martone ME, Mons A, Packer AL, Persson B, Rocca-Serra P, Roos M, van Schaik R, Sansone S, Schultes E, Sengstag T, Slater T, Strawn G, Swertz MA, Thompson M, van der Lei J, van Mulligen E, Velterop J, Waagmeester A, Wittenburg P, Wolstencroft K, Zhao J, Mons B (2016). The FAIR Guiding Principles for scientific data management and stewardship. Sci Data.

[385] Boaz A, Hanney S, Borst R, O'Shea A, Kok M (2018). How to engage stakeholders in research: design principles to support improvement. Health Res Policy Syst.

[386] Michie S, West R, Finnerty AN, Norris E, Wright AJ, Marques MM, Johnston M, Kelly MP, Thomas J, Hastings J (2020). Representation of behaviour change interventions and their evaluation: development of the Upper Level of the Behaviour Change Intervention Ontology. Wellcome Open Res.

[387] Leslie M, Eales J, Fast J, Mortenson W, Atoyebi O, Khayatzadeh-Mahani A (2019). Towards sustainable family care: using goals to reframe the user-centred design of technologies to support carers. Int J Care Caring.

[388] Harrington RA, Gray M, Jani A (2020). Digitally enabled social prescriptions: adaptive interventions to promote health in children and young people. J R Soc Med.

[389] Jani A, Gray M (2019). Making social prescriptions mainstream. J R Soc Med.

[390] Plouffe LA, Garon S, Brownoff J, Eve D, Foucault ML, Lawrence R (2013). Advancing age-friendly communities in Canada. Can Rev Social Policy/Revue canadienne de politique sociale.

[391] Poveda-Villalón M, Espinoza-Arias P, Garijo D, Corcho O (2020). Coming to terms with FAIR ontologies. Knowledge Engineering and Knowledge Management.

[392] Pinto HS, Martins J (2000). Reusing Ontologies.

[393] Dooley DM, Griffiths EJ, Gosal GS, Buttigieg PL, Hoehndorf R, Lange MC, Schriml LM, Brinkman FS, Hsiao WW (2018). FoodOn: a harmonized food ontology to increase global food traceability, quality control and data integration. NPJ Sci Food.

