# Supplementary material for: Social Prescription Interventions Addressing Social Isolation and Loneliness in Older Adults: Meta-Review Integrating On-the-Ground Resources
Source: J Med Internet Res. 2023 May 17;25:e40213. doi: 10.2196/40213 (PMC10233446; doi:10.2196/40213)
Supplement: Multimedia Appendix 1 [file jmir_v25i1e40213_app1.docx]

**Appendix 1.** Search terms

| **Older population terms**  **(full text search)** | **Social isolation and loneliness**  **(full text search)** | **Review related terms**  **(limited to title and abstract search)** |
| --- | --- | --- |
| Older*  (Old* age*)  Elder*  Senior*  Aged  Ag?ing  Geriatric  (Old people)  (Oldest old) | (Social* isolat*)  Lonel*  (Social* participat*)  (Social* integrat*)  (Social* connect*)  (Social network*)  (Social* support*)  (Social* interact*) | Review *[limited to title search]*  (Systematic Review)  (Meta-review)  (Meta-analysis)  (Scoping review)  (Rapid review) |
