# Supplementary material for: Social Prescription Interventions Addressing Social Isolation and Loneliness in Older Adults: Meta-Review Integrating On-the-Ground Resources
Source: J Med Internet Res. 2023 May 17;25:e40213. doi: 10.2196/40213 (PMC10233446; doi:10.2196/40213)
Supplement: Multimedia Appendix 2 [file jmir_v25i1e40213_app2.docx]

**Appendix 2.** List of reviews included with types of interventions reviewed*.*

| **Review** | **Years reviewed** | **Relevant studies** | **Interventions Outcomes** | | | | **Quality Appraisal: AMSTAR Questions Satisfied** |
| --- | --- | --- | --- | --- | --- | --- | --- |
|  |  |  | **Increasing social interactions** | **Providing instrumental support** | **Promoting mental & physical well-being** | **Improving social (health) care** |  |
| Abdi et al. (2018) [54] | 2000-2017 | 5 |  | AT(3) | NC(2) |  | Q3, Q4, Q5, Q8, Q9, Q13 |
| Bemelmans et al. (2012) [55] | 2002-2012 | 1 |  | AT(1) |  |  | Q3, Q4, Q5, Q8, Q9, Q13 |
| Buyl et al. (2020) [56] | 2000-2018 | 2 | ST(2) |  |  |  | Q4, Q5, Q6, Q8, Q9 |
| Campos et al. (2019) [57] | 2001-2017 | 4 | ST(1) | AT(3) |  |  | Q3, Q4, Q5, Q6, Q8, Q9, Q13 |
| Cattan et al. (2005) [47] | 1970-2002 | 19 | CC(1), ST(3) | AT(1) | PT(2), PA(2) | TH(2), HS(8) | Q3, Q4, Q5, Q13 |
| Chao et al. (2015) [58] | 2006-2013 | 2 |  |  | PA(2) |  | Q4, Q8 |
| Chen & Schulz (2016) [52] | 2002-2015 | 12 | ST(11) |  | NC(1) |  | Q3, Q4, Q5, Q6, Q8, Q9 |
| Chen et al. (2018) [59] | 2000-2017 | 1 |  |  | NC(1) |  | Q3, Q4, Q5, Q6, Q8 |
| Chipps et al. (2017) [60] | 2000-2017 | 7 | ST(5) |  | PA(1), RA(1) |  | Q3, Q4, Q5, Q6, Q8, Q13 |
| Choi et al. (2012) [53] | 2001-2012 | 5 | ST(5) |  |  |  | Q4, Q8, Q9 |
| Cohen-Mansfield & Perach (2015) [51] | 1996-2011 | 20 | IG(1), CC(5), ST(6) | AT(1), OT(1) | PT(1), PA(2), RA(3) |  | Q3, Q8 |
| Dickens et al. (2011) [49] | <2009 | 17 | IG(2), CC(3), ST(5) |  | PT(3), RA(2) | TH(2) | Q3, Q4, Q5, Q6, Q8, Q9, Q13 |
| Forsman et al. (2011) [61] | no time limit | 4 |  |  | PA(3), RA(1) |  | Q4, Q5, Q8, Q9, Q13 |
| Forsman et al. (2018) [62] | 2003-2014 | 10 | ST(7) | AT(1) | RA (2) |  | Q4, Q5, Q8 |
| Franck et al. (2016) [63] | 2009-2013 | 4 | ST(1) |  | RA (3) |  | Q3, Q4, Q5, Q6, Q8, Q9, Q13 |
| Galbraith et al. (2015) [64] | n.r. | 5 | IG(5) |  |  |  | Q4, Q5 |
| Gardiner et al. (2018) [48] | 2003-2016 | 19 | IG(1), CC(6), ST(4) |  | PT(3), RA(3) | HS(2) | Q3, Q4, Q5, Q8, Q13 |
| Gee & Mueller (2019) [65] | 1965-2018 | 3 |  |  | NC(3) |  | Q3, Q4, Q8 |
| Gerritzen et al. (2019) [66] | 2009-2017 | 1 | IG(1) |  |  |  | Q4, Q5, Q6, Q8 |
| Gualano et al. (2018) [67] | n.r. | 4 | IG(4) |  |  |  | Q3, Q4, Q5, Q6, Q8, Q9, Q13 |
| Hagan et al. (2014) [68] | 2000-2012 | 8 | CC(6) |  | PT(2) |  | Q4, Q8 |
| Heaven et al. (2013) [69] | n.r. | 7 | IG(6), CC(1) |  |  |  | Q4, Q5, Q8, Q9, Q13 |
| Jarvis et al. (2020) [70] | 2000-2017 | 13 | IG(1), CC(3), ST(4) |  | PT(1), PA(2), RA(1) | HS(1) | Q3, Q4, Q5, Q8 |
| Kachouie et al. (2014) [71] | n.r. | 3 |  | AT(3) |  |  | Q3, Q4, Q5, Q6, Q8, Q9, Q13 |
| Khosravi & Ghapanchi (2016) [72] | 2000-2015 | 1 |  | AT(1) |  |  | Q3, Q4, Q5 |
| Knight et al. (2014) [73] | 1990-2012 | 5 | IG(5) |  |  |  | Q4, Q8 |
| Koo & Vizer (2019) [74] | <2018 | 2 |  | AT(2) |  |  | Q3, Q4, Q5, Q6, Q8, Q13 |
| Lee et al. (2020) [75] | 1997-2018 | 7 | IG(7) |  |  |  | Q3, Q4, Q8 |
| Li et al. (2018) [76] | <2017 | 5 |  |  | PA(5) |  | Q4, Q8, Q9 |
| Masi et al. (2011) [77] | 1970-2009 | 14 | CC(4), ST(5) |  | PT(1), PA(1), RA(1) | TH(2) | Q3, Q4, Q5, Q8, Q13 |
| Medical Advisory (2008) [78] | 1980-2008 | 11 | CC(1), ST(1) | AT(1) | PT(2), PA(2), RA(1) | TH(3) | Q4, Q8 |
| Morris et al. (2014) [79] | 2000-2013 | 4 | ST(4) |  |  |  | Q4, Q5, Q8, Q9, Q13 |
| Nicholas et al. (2019) [80] | 2008-2018 | 1 |  |  | RA(1) |  | Q3, Q4, Q8 |
| Noice et al. (2014) [81] | n.r. | 15 |  |  | RA(15) |  | Q3, Q4, Q5, Q8 |
| Papageorgiou et al. (2016) [50] | 2005-2015 | 10 | IG(1), CC(2), ST(3) | OT(2) |  |  | Q4, Q6, Q8, Q9, Q13 |
| Pool et al. (2017) [82] | n.r. | 2 | CC(1) |  | PA(1) |  | Q4, Q5, Q8, Q9 |
| Portz (2017) [83] | n.r. | 3 | ST(1) |  |  | TH(2) | Q3, Q4, Q8, Q13 |
| Poscia et al. (2018) [38] | 2011-2016 | 19 | IG(2) CC(6), ST(1) | AT(1), OT(2) | PA(1), RA(6) |  | Q4, Q5, Q8, Q9, Q13 |
| Pu et al. (2019) [84] | n.r. | 3 |  | AT(1) | NC(2) |  | Q3, Q4, Q5, Q8, Q9, Q13 |
| Roets-Merken et al. (2015) [85] | <2014 | 1 |  | OT(1) |  |  | Q4, Q5, Q6, Q8, Q9, Q13 |
| Sadarangani & Murali (2018) [86] | 1987-2017 | 1 |  |  | RA(1) |  | Q4, Q8 |
| Shishehgar et al. (2018) [87] | 2000-2015 | 2 |  |  | NC(2) |  | Q3, Q4 |
| Shvedko et al. (2018) [88] | <2017 | 7 |  |  | PA(7) |  | Q3, Q4, Q5, Q6, Q8, Q9, Q13 |
| Sims-Gould et al. (2017) [89] | n.r. | 2 |  | OT(2) |  |  | Q4, Q5, Q8, Q9, Q13 |
| Smallfield & Molitor (2018) [90] | 1995-2015 | 6 | CC(1) | OT(1) | PT(2), RA(2) |  | Q4, Q5, Q6, Q9, Q13 |
| Turcotte et al. (2018) [91] | 1995-2017 | 4 | ST(1) | OT(5) |  |  | Q3, Q4, Q5, Q8 |
| Veazie et al. (2019) [92] | 2013-2018 | 10 | IG(1) | OT(3) | PA(2), RA(4) |  | Q4, Q6, Q8, Q9, Q13 |
| Virues-Ortega et al. (2012) [93] | 1985-2008 | 3 |  |  | NC(3) |  | Q3, Q4, Q5, Q8, Q9, Q13 |
| Wang et al. (2013) [94] | n.r. | 2 |  |  | RA(2) |  | Q3, Q4, Q5, Q6, Q8 |
| Zhang et al. (2016) [95] | n.r. | 2 | IG(2) |  |  |  | Q4, Q8 |

*N.r: not reported; ST: Social technology; IG: Intergenerational interventions; CC: Conducive communities; OT: Occupational therapy/rehab; AT: Assistive technology; NC: Non-human companions; RA: Recreational activities; PT: Psychological therapy; PA=Physical activity; HS: Home-based health services; TH: Telehealth.*
