# Supplementary material for: Social Prescription Interventions Addressing Social Isolation and Loneliness in Older Adults: Meta-Review Integrating On-the-Ground Resources
Source: J Med Internet Res. 2023 May 17;25:e40213. doi: 10.2196/40213 (PMC10233446; doi:10.2196/40213)
Supplement: Multimedia Appendix 3 [file jmir_v25i1e40213_app3.docx]

**Appendix 3.** Interventions and effectiveness

| **Intervention Theme and Subthemes** | | **Studies Overview** | **Intervention Subcategories** | **Intervention Context** | **Outcomes*** |
| --- | --- | --- | --- | --- | --- |
|  |  |  |  |  |  |
| **Increasing social interactions** | *Social technology* | 27 studies  ***N*_Total_***=* 1700  ***N*_Range_***=* 4 – 205  33% RCT  26% Quasi-exp.  22% Mixed  15% Qual.  4% Pilot | 59% ICT training  26% Telephone and video calling befriending initiatives  11% Internet social network tools  4% Radio programming | **Site:** home (e.g., private residence, retirement village, low-income housing), day care-centres and community college  **Facilitator:** peer tutors, trained ICT professionals and educators, computer consultants, occupational therapists, nurses, researchers  26% group  22% individual  22% mixed | 33% Loneliness  19% Social network  17% Depression |
|  |  |  |  |  |  |
|  |  |  |  |  |  |
|  |  |  |  |  |  |
|  | *Intergenerational interventions* | 27 studies  ***N*_Total_***=* 1588  ***N*_Range_** *=* 5 - 180  56% Quasi-exp  15% Qual  11% Mixed  11% Pilot  7% RCT | 44% Recreational activities, arts and/or games  22% Education and skills development  15% Reading activities  11% Therapy-focused activities  7% Mentorship | **Site:** senior centres, adult day care centres, schools  **Facilitator:** social care professionals, on-site staff  89% group  11% individual | 20% Social support  17% Social network  17% Depression  14% Other soc. health  11% Anxiety |
|  |  |  |  |  |  |
|  |  |  |  |  |  |
|  |  |  |  |  |  |
|  | *Conducive communities* | 22 studies  ***N*_Total_***=* 3431  ***N*_Range_***=* 5 – 817  45% Quasi-exp  36% RCT  9% Pilot  5% Qual.  5% Mixed | 23% Social groups  23% Support groups  23% Social service-focused  18% Education-based support groups  14% Peer mentoring/befriending | **Site:** community centres, day care centres, senior centres  **Facilitator:** professional facilitators, peer educators, volunteers, on-site staff, social workers  68% group  18% individual  9% mixed | 32% Loneliness  19% Social support  15% Social isolation |
|  |  |  |  |  |  |
|  |  |  |  |  |  |
|  |  |  |  |  |  |
|  |  |  |  |  |  |
|  |  |  |  |  |  |
|  |  |  |  |  |  |
| **Promoting mental and physical well-being** | *Non-human companions* | 12 studies  ***N*_Total_***=* 416  ***N*_Range_***=* 9 – 94  42% RCT  33% Quasi-exp  17% Qual.  8% Pilot | 50% Animal-assisted  50% Robo-pets | **Site:** home, lab-based, day care centre  **Facilitator:** certified volunteers, researchers  8% group  75% individual | 35% Depression  29% Anxiety  29% Loneliness |
|  |  |  |  |  |  |
|  |  |  |  |  |  |
|  |  |  |  |  |  |
|  | *Recreational activities* | 39 studies  ***N*_Total_***=* 2156  ***N*_Range_***=*  41% Quasi-exp  23% RCT  18% Qual.  10% Pilot  8% Mixed | 31% Music classes and performances  23% Social activity group  18% Fine arts  13% Nature  8% Theater classes  8% Video games | **Site:** home, day service centre  **Facilitator:** trained artists and educators (e.g., musicians, dancers, actors), staff educators, researcher  95% group  3% individual  3% mixed | 23% Mental well-being  22% Depression  17% Loneliness  14% Social isolation |
|  |  |  |  |  |  |
|  |  |  |  |  |  |
|  |  |  |  |  |  |
|  | *Psychological therapy* | 6 studies  ***N*_Total_***=* 938  ***N*_Range_***=* 26 - 339  67% RCT  33% Quasi-exp | 100% Group-based therapy | **Site:** Senior centers  **Facilitator:** social workers, psychologists, nurses, peer facilitators, counsellors | 31% Loneliness  15% Depression |
|  |  |  |  |  |  |
|  |  |  |  |  |  |
|  |  |  |  |  |  |
|  | *Physical activity* | 23 studies  ***N*_Tota_**_l_= 3729  ***N*_Range_**= 7 - 849  65% RCT  17% Pilot  13% Quasi-exp  4% Mixed | 30% Exergames  22% Specialized muscle or aerobic exercise programs  17% Exercise and education  13% Martial arts  13% Walking routines  4% Exercise-driven rehabilitation | **Site:** Care retirement community, activity centre, rehabilitation centre,  **Facilitator:** trained sports/exercise instructors, peers, trained staff  78% group  4% individual  9% mixed | 32% Loneliness  23% Social network  19% Social support  19% Depression |
|  |  |  |  |  |  |
|  |  |  |  |  |  |
|  |  |  |  |  |  |
| **Providing instrumental support** | *Occupational therapy/ rehabilitation* | 15 studies  ***N*_Total_***=* 2582  ***N*_Range_***=* 28-460  67% RCT  13% Quasi-exp  13% Pilot  7% Mixed | 40% Wellness education  33% Holistic lifestyle programs  13% Individualized treatment plans  13% OT-based Recreational activities | **Site:** home, rehabilitation centre  **Facilitator:** occupational and physiotherapists, rehabilitation counselor, home care aides, allied professionals, nurses, social workers  20% group  20% individual  40% mixed | 27% Mental well-being  23% Depression  12% Loneliness  12% Social support |
|  | *Assistive technology* | 16 studies  ***N*_Total_***=* 621  ***N*_Range_***=* 1 - 140  38% Quasi-exp  31% Qual.  25% Pilot  6% RCT | 63% Assistive robots  19% Assistive technology devices  19% sensory technology | **Site:** health service facility, day service or care centre, home  **Facilitator:** nurses, researchers, physicians  38% group  13% individual  19% mixed | 31% Mental well-being  27% Social communication/ participation  19% Depression |
| **Providing home and community care** | *Home-based health services* | 10 studies  **N_Total_**= 3748  **N_Range_**= 39 - 580  60% RCT  40% Quasi-exp | 50% At-home visits  20% Screening for social isolation  20% Combined screening, referrals, and home visits  10% Hospital-to-home transition | **Site:** home, hospital-to-home  **Facilitator:** nurses, physicians, social workers, volunteers, trained “gatekeepers”, psychology students  80% individual | 53% Loneliness  47% Social isolation |
|  |  |  |  |  |  |
|  |  |  |  |  |  |
|  |  |  |  |  |  |
|  | *Telehealth* | 8 studies  ***N*_Total_** *=* 686  **N_Range_***=* 61 - 106  63% RCT  25% Quasi-exp  13% Pilot | 63% Phone-based  38% Web-based (including online health information) | **Site:** home  **Facilitator:** clinicians, social workers, counsellors, nurses  38% group  63% individual | 36% Depression  29% Loneliness  14% Social isolation |
|  |  |  |  |  |  |
|  |  |  |  |  |  |
|  |  |  |  |  |  |

** indicates the specific social and mental health outcomes that comprise greater than 10% of total outcomes reported for each intervention subtheme.*
